# The absence of association between anorexia nervosa and smoking: converging evidence across two studies

**DOI:** 10.1007/s00787-021-01918-z

**Published:** 2021-12-22

**Authors:** E. Caitlin Lloyd, Zoe E. Reed, Robyn E. Wootton

**Affiliations:** 1 Department of Psychiatry, Columbia University Irving Medical Center, New York, NY USA; 2 New York State Psychiatric Institute, New York, NY USA; 3 School of Psychological Science, University of Bristol, Priory Road, Bristol, UK; 4 MRC Integrative Epidemiology Unit, University of Bristol, Bristol, UK; 5 Nic Waals Institute, Lovisenberg Diaconal Hospital, Oslo, Norway

**Keywords:** Anorexia nervosa, Epidemiology, Longitudinal, Mendelian randomization, Smoking, Triangulation

## Abstract

Previous studies have found increased smoking prevalence amongst adults with anorexia nervosa (AN) compared to the general population. The current investigation explored bidirectional associations between AN and smoking behaviour (initiation and heaviness), to address questions surrounding causation. In Study One, logistic regression models with variance robust standard errors assessed longitudinal associations between AN and smoking, using data from adolescent participants of the Avon Longitudinal Study of Parents and Children (*N* = 5100). In Study Two, two-sample Mendelian randomisation (MR) tested possible causal effects using summary statistics from publicly available genome-wide association studies (GWAS). Study One provided no clear evidence for a predictive effect of AN on subsequent smoking behaviour, or for smoking heaviness/initiation predicting later AN. MR findings did not support causal effects between AN and smoking behaviour, in either direction. Findings do not support predictive or causal effects between AN and smoking behaviour. Previously reported associations may have been vulnerable to confounding, highlighting the possibility of smoking and AN sharing causal risk factors.

**Supplementary Information:**

The online version contains supplementary material available at 10.1007/s00787-021-01918-z.

## Introduction

Anorexia nervosa (AN) is an eating disorder characterised by persistent food restriction and an abnormal attitude towards eating and weight (e.g., distorted self-perception, fear of eating and weight-gain; [1]). AN has one of the highest mortality rates of all psychiatric disorders [2], and severe effects on physical and mental health that last beyond weight recovery [3–6]. Amongst adults with AN, tobacco smoking and nicotine dependence are reported as elevated as compared with healthy controls (HC; [7–9]). Amongst adolescents, it is commonly reported that weight concern, one of the core psychological symptoms of AN, is associated with increased risk of current and future smoking [10, 11]. There is some evidence to support nicotine suppressing appetite and increasing metabolism [12], and individuals with AN cite motivators for smoking to include weight-loss/avoidance of eating [13]. Such observations lend support to the proposal that AN pathology, (or the drive to maintain a low body weight), causes smoking (e.g. [14]).

The observed association between AN and smoking could, however, be explained by causal effects in the reverse direction. For example, appetite suppressing effects of smoking may instigate food restriction and weight loss that is experienced as reinforcing by those with a vulnerability to AN, prompting further and excessive engagement in restrictive eating behaviour (e.g. [15–18]). Equally, smoking may increase AN risk by affecting brain structure and function (e.g. [19, 20]). Finally, AN and smoking might not be causally related at all and could instead share causal risk factors: confounding could induce an association between the two.

Smoking causes various noncommunicable diseases and is the leading cause of preventable death globally [21]. Understanding how smoking and AN development are associated may thus inform mechanisms underpinning the disproportionate burden of disease amongst psychiatric patients [22], as well as AN aetiology. Though previous studies have explored the co-occurrence of AN and smoking, they have been limited in their ability to make causal inferences. To our knowledge, no study has probed the longitudinal association between AN and smoking. Yet, to establish that one phenotype (a) causes another (b) it is necessary, though not sufficient, to demonstrate that phenotype (a) precedes phenotype (b). Furthermore, observational research (both cross-sectional and longitudinal) is prone to bias arising from confounding and reverse causation. Confidence in outcomes of observational research may be strengthened by triangulation with outcomes of studies of differing design, given that each design will be vulnerable to different sources of error [23].

In the current investigation we study the association between AN and smoking using two designs that have not yet been applied to this research question. In Study One, we conduct observational longitudinal analyses addressing bidirectional associations between AN and smoking in a large adolescent population sample. The adolescent period is particularly relevant for addressing questions surrounding temporal patterns of smoking and AN occurrence, given AN onset is typically between the ages of 15 and 19 [24], and smoking also typically starts during adolescence [25]. To date, and to our knowledge, no study has specifically studied smoking behaviour amongst adolescents with AN or observational associations between smoking and AN in an adolescent cohort.

Study Two adopts a two-sample Mendelian randomization (MR [26]) design. MR employs genetic variants as instruments for an exposure of interest, minimising bias due to confounding and reverse causation [27]. Both AN and smoking behaviours are moderately heritable, with twin studies suggesting 50–60% of the variance in AN and 44–75% of the variance in smoking behaviours is explained by genetic factors [28, 29]. A reasonable proportion is explained by common genetic variants, with single nucleotide polymorphism (SNP) heritability estimates of 11–17% for AN and up to 8% for smoking behaviours [30, 31]. Current genetic instruments for AN and smoking explain 1.7% and 2.3% of the variance, respectively [30, 31]. Given the different sources of bias from each study design, consistent inferences across Studies One and Two would thus increase confidence in the validity of the results. Given previous findings concerning the presence and motivators of smoking behaviour amongst AN, and support for nicotine affecting body weight and neural function in ways that could conceivably increase risk for AN, we hypothesised causal effects in both directions.

### Methods and materials

#### Study one

##### Data sources

To complete the longitudinal observational analysis, data from the Avon Longitudinal Study of Parents and Children (ALSPAC [32–34]) was used. ALSPAC is a prospective population cohort study that initially recruited 14,541 mothers living in Avon, UK, whose expected delivery dates were between 1st April 1991 and 31st December 1992. Further eligible mothers have since been recruited, and the total sample comprises 15,454 pregnancies, with 14,901 children alive at one year. The ALSPAC study website provides details of all available data, through a fully searchable data dictionary and variable search tool. Ethics approval for the study was obtained from the ALSPAC Ethics and Law Committee and local Research Ethics Committees.

**Fig. 1:**
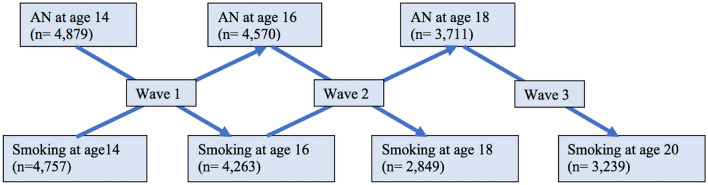
Diagram of longitudinal waves of analysis and available participant data for the current study

The current analysis assessed the longitudinal association between AN and subsequent smoking behaviours (heaviness and initiation), and vice versa, across multiple waves of data. Consenting participants who had the required smoking and AN data, in either direction of interest, at any given wave (Fig. 1), were included providing baseline data (collected at age 14) for the particular outcome was also available (*n* = 5100).

##### Measures

*Smoking* Smoking behaviour was assessed annually when adolescents were between the ages of 13 and 18, and again at age 20. In the current study, smoking behaviour at ages 14, 16, 18 and 20 were variables of interest; however, data from other time-points were used for classification. In particular, smoking behaviour at each time-point of interest was recorded as the heaviest of that time-point and the one the year prior. The smoking heaviness variable was classified as none, occasional, weekly, or daily smoking, using previously applied criteria [35] detailed in Table S1. A second smoking variable, smoking initiation, was derived given associations between AN and beginning to smoke may differ from associations between AN and smoking heaviness. Smoking initiation at each time point was defined as meeting criteria for daily or weekly smoking at the time-point of interest (or the one the year earlier), when not meeting these criteria at prior time-points, and not reporting smoking more than 3 times in the prior 6 months at age 10 (assessed using self-report questionnaires completed during clinic visits). The smoking initiation variable was dichotomized to have the possible values of weekly/daily smoking or no smoking, as it was relatively rare to meet criteria for weekly or daily smoking initiation and AN at any wave.

*AN* AN was assessed at age 14, 16, 18 and recorded when participants met DSM-5 diagnostic criteria for the disorder, based on previously defined thresholds [36] described in the Supplement (Table S2).

*Covariates* Variables that may confound the association between AN and smoking were identified from literature and theory surrounding risk factors for the two outcomes. These were sex, socio-economic status (a binary variable based on occupations of both parents), mother parity (a binary indicator of whether mothers had previous viable pregnancies), mother age at delivery, mother lifetime AN, mother smoking during the final two months of pregnancy, child body mass index (BMI) *z*-score at age 10 and child worry at age 10. For measure details see the Supplement.

To avoid introducing bias when data are analysed under a complete case approach, predictors of missingness should be included in analytical models [37, 38]. In ALSPAC, socio-economic status, mother parity and mother age at delivery are predictors of missingness [32], further justifying inclusion of these covariates in analysis models.

##### Statistical analysis

Statistical analyses were conducted using Stata15 [39]. Ordinal logistic regression models (unadjusted and adjusted for potential confounders) assessed prospective associations between AN and subsequent smoking heaviness, across three waves of data (Fig. 1). To account for the clustering in repeated measures data, or the non-independence of observations from the same participant, variance-robust standard errors were calculated. Generalised estimating equations (GEEs) assessed the association between AN and subsequent smoking initiation (the binary variable that comprised the second smoking variable). In this analysis, participant data were not included at a given wave if the participant had initiated smoking on a weekly or daily basis at an earlier wave.

GEEs also estimated the association between smoking behaviour exposures (heaviness and initiation) and subsequent AN development, across two longitudinal waves (Fig. 1). Participants were not included in the analysis concerning predictive effects of smoking initiation at a particular wave if they had reported the initiation of smoking at an earlier wave. The GEEs specified a logit link function, a binomial outcome distribution and an unstructured correlation within repeated measures outcome data. Robust standard errors were calculated. GEE analyses were completed both unadjusted and adjusted for all potential confounders. All fully adjusted GEE and regression models of the current study included wave as a covariate.

We conducted complete case, maximum available, and imputed data analyses. In complete case analyses, we included only participants who had data for all variables of the fully adjusted statistical models in both unadjusted and adjusted analyses. In maximum available data analyses, participants were included in unadjusted analyses provided they had data for the variables in the unadjusted model, and in the adjusted model when they had data for all variables of the adjusted model. In imputed data analyses, participants were included providing they had data for the unadjusted analyses, and missing covariate data were imputed using a multiple imputation by chained equation (MICE) approach. To leverage as much information as possible in the prediction of missing covariate data, the imputed datasets were created using the entire ALSPAC sample (*n* = 14,901). The imputation model included all analysis variables and ethnicity—to improve prediction. In total, 50 datasets including complete covariate data were imputed. To complete analyses with imputed data, the statistical models were applied in each of the 50 imputed datasets, and effect estimates from the 50 analyses averaged to derive the magnitude of association between the outcome and explanatory variables. In imputed data analysis, standard errors surrounding effect estimates account for the variance caused by the sampling method, variance arising from the missing data, and an additional source of variance resulting from the imputation procedure itself [40]. Observed and imputed data for all covariates is presented in Table S3. Results from our imputed data analyses are emphasised given the improved efficiency of this approach, and preservation of relevant exposure and outcome data [37]. However, full results of complete case and maximum available analyses are also reported.

For comparison with longitudinal outcomes, cross-sectional analyses were completed. Here, smoking heaviness and initiation variables were regressed onto AN assessed at the same time point (at ages 14, 16, 18), using the modelling procedures described for longitudinal analyses.

### Study 2

#### Methods and materials

In Study 2, we use two-sample (or summary level) Mendelian randomisation (MR) to test bidirectional causal effects between AN and smoking behaviours. MR employs genetic variants as proxies for an exposure of interest (e.g. AN) to test a causal effect of that exposure on an outcome (e.g. smoking [26]). This method takes advantage of the random and independent inheritance of genetic variants at conception to minimise risk of bias due to confounding. As the outcome cannot influence inherited DNA, bias due to reverse causation is also reduced.

MR makes the following three crucial assumptions: (1) the genetic instrument is robustly associated with the exposure, (2) the genetic variant is not associated with confounders and (3) the genetic variants are associated with the outcome only through the exposure. To meet the first assumption, we use only genome-wide significant genetic variants from the largest (and best powered) GWAS for each exposure in our primary analyses. To test the latter two assumptions, we conduct several sensitivity analyses able to detect pleiotropy, which occurs when one genetic variant has effects on multiple phenotypes. Pleiotropy can violate assumptions 2 or 3 if the other phenotypes are not on the causal pathway between smoking and AN. The two-sample MR method uses summary statistics from previously conducted, and independent, GWAS to estimate causal effects [23].

##### Data sources

Details of GWAS summary statistics used in the current analysis are presented in Table 1. To derive each instrument for use in the MR analyses, SNPs were clumped for independence at *r*^2^ < 0.001 and a LD window of 10000 kb using the *clump_data* command from the TwoSampleMR package, which uses the 1000 Genomes LD reference panel [41].

**Table 1:** GWAS Summary Statistics used in two-sample MR analysis

Phenotype	References	Sample	N SNPs*	Ancestry	GWAS N	Phenotype definition
Anorexia Nervosa	[31]	PGC Consortium	8	European	16,992 cases 55,525 controls	Lifetime diagnosis of AN via hospital or register records, structured clinical interviews or online questionnaires based on DSM/ICD criteria. UK Biobank self-reported a diagnosis
Smoking Initiation	[30]	GSCAN Consortium	203	European	1,232,091 as an exposure and 632,802 as an outcome (23andMe removed due to data access restrictions)	Ever having smoked more than 100 cigarettes or ever having smoked regularly
Lifetime Smoking	[77]	UK Biobank	126	European	462,690	Composite of smoking initiation, duration, heaviness and cessation

*PGC* Psychiatric Genomics Consortium, *GSCAN* GWAS & Sequencing Consortium of Alcohol and Nicotine use

^*^N SNPs refers to the number of available SNPs at the genome-wide level of significance following the clumping procedure described above to ensure independence

##### Statistical analysis

All analyses were conducted in R [42], using the TwoSampleMR package [41]. There were 8 instrumental SNPs for AN, 203 for smoking initiation and 126 for lifetime smoking. Due to the low number of SNPs associated with AN at the genome-wide significance threshold (which reduces power in MR analyses), we conducted sensitivity analyses using a relaxed threshold of *p* < 5 × 10^–5^ for instrument identification. For each genetic instrument we estimated the *F*-statistic as a test of instrument strength, to check for possible weak instrument bias [43]. We used the two-sample approximation outlined in Bowden et al. [43], calculating the *F*-statistic for each SNP independently. We present the mean of the *F*-statistics; *F* > 10 indicates that weak instrument bias is unlikely.

For each analysis, the association of each exposure SNP with the outcome was identified in an independent GWAS of the outcome. When SNPs were not available in the outcome GWAS, the TwoSampleMR package attempts to find proxies at LD *r*^2^ threshold of > 0.8, using the 1000 Genomes project as a reference panel [41]. Where this failed, manual attempts to identify proxy variants were made (specifying the same LD *r*^2^ threshold of > 0.8), using the package proxysnps [44]. Proxies replaced original variants for instrument-exposure and instrument-outcome analyses. Palindromic SNPs were aligned if the minor allele frequency was below 0.3.

The primary analysis was the inverse-variance weighted (IVW) method, which assumes that all instruments are valid and does not include an intercept in the model [45]. Therefore, invalid instruments will bias the IVW estimate unless pleiotropy is balanced. Three additional methods that each make different assumptions about pleiotropy were also conducted: MR Egger [46]; weighted median [47]; and weighted mode [48] analyses. The strongest evidence for a causal effect would be a consistent estimate across all four methods. The MR Egger method allows for directional pleiotropic effects, such that some SNPs could be acting on the outcome via a pathway that does not involve the exposure. The intercept is not constrained to zero and provides an estimate of the directional pleiotropic effect [46]. The weighted median approach assumes that at least 50% of the total weight of the instrument comes from valid variants [47]. The weighted mode approach assumes that the most common causal effect is consistent with the true causal effect [48]: the remaining instruments could be invalid (i.e., violating the assumptions of MR) without biasing the estimated causal effect.

Cochran’s Q test of heterogeneity across SNP effects was completed to inform the validity of conclusions arising from the IVW estimate, since consistent estimates across SNPs are unlikely in the absence of a true underlying causal effect. The MR Egger intercept test was also conducted, where evidence of a deviation from the origin would be expected if directional horizontal pleiotropy was present [46].

To determine the suitability of the MR Egger test, we estimated regression dilution (caused by measurement error in the instrument-exposure associations) in the MR Egger analysis, by calculating the $I_{{{\text{GX}}}}^{2}$ statistic [43]. Where $I_{{{\text{GX}}}}^{2}$ was greater than 0.9, MR Egger was conducted. If it was between 0.6 and 0.9, a simulation extrapolation correction was applied to the MR Egger estimate. $I_{{{\text{GX}}}}^{2}$ values below 0.6 indicate that MR Egger tests are not suitable; where this was the case we do not present MR Egger estimates of causal effect and corresponding estimates of pleiotropic effects should be interpreted cautiously [43].

To visualize results, we present scatter plots of SNP-exposure effects by SNP-outcome effects. We conducted leave-one-out analyses and calculated single-SNP Wald ratios as sensitivity analyses. Forest plots of these results and further details are presented in the Supplementary Materials. We conducted a power analysis using the online power calculator for Mendelian randomisation (https://shiny.cnsgenomics.com/mRnd/; [49]). For further details see the Supplement (Table S4).

Websites accessed: http://www.bristol.ac.uk/alspac/researchers/our-data/, https://www.med.unc.edu/pgc/download-results/)

### Results

#### Study one

##### Sample characteristics

Details of participant demographic characteristics and data provision are given in the Supplement (Table S5 and Fig. S1 respectively). The Supplement also details the distribution of smoking and AN by gender (Table S5).

##### Prospective prediction of smoking by anorexia nervosa

In unadjusted analyses, there was no support for AN predicting smoking heaviness (see Table 2). Outcomes of adjusted analyses were consistent. Similarly, there was no strong evidence to support an association between AN and smoking initiation in unadjusted or adjusted analyses.

In unadjusted analyses there was no strong statistical support for the association between smoking heaviness and subsequent AN (see Table 2). In adjusted analyses there was similarly no strong evidence for an association. There was no clear evidence for an association between smoking initiation and subsequent AN development in unadjusted, or adjusted, analyses.

**Table 2:** Results of Longitudinal Analyses

	Association between Smoking Heaviness and prior Anorexia Nervosa						Association between Anorexia Nervosa and prior Smoking Heaviness					
Data^a^	Unadjusted			Adjusted^b^			Unadjusted			Adjusted^b^		
	OR [95% CI]	*P*	*N*	OR [95% CI]	*P*	*N*	OR [95% CI]	*P*	*N*	OR [95% CI]	*P*	*N*
Complete case	1.76 [0.78, 3.94]	0.17	2968	1.5 [0.63, 3.54]	0.36	2968	1.35 [0.94, 1.94]	0.10	3339	1.55 [1.04, 2.31]	0.03	3339
Maximum available	1.41 [0.72, 2.74]	0.32	4245	1.5 [0.63, 3.54]	0.36	2968	1.16 [0.82, 1.65]	0.40	4597	1.55 [1.04, 2.31]	0.03	3339
Imputed	1.41 [0.72, 2.74]	0.32	4245	1.25 [0.6, 2.59]	0.55	4245	1.16 [0.82, 1.65]	0.40	4597	1.27 [0.85, 1.89]	0.25	4597

	Association between Smoking Initiation and prior Anorexia Nervosa						Association between Anorexia Nervosa and prior Smoking Initiation					
	Unadjusted			Adjusted^b^			Unadjusted			Adjusted^c^		
	OR [95% CI]	*P*	*N*	OR [95% CI]	*P*	*N*	OR [95% CI]	*P*	*N*	OR [95% CI]	*P*	*N*
Complete case	0.76 [0.24, 2.44]	0.65	975	0.91 [0.2, 4.25]	0.91	975	1.06 [0.26, 4.34]	0.94	2850	1.59 [0.25, 10.34]	0.63	2914
Maximum available	0.70 [0.23, 2.08]	0.52	1226	0.91 [0.2, 4.25]	0.91	975	0.78 [0.21, 2.9]	0.72	3610	1.59 [0.25, 10.34]	0.63	2914
Imputed	0.70 [0.23, 2.08]	0.52	1226	0.78 [0.17, 3.64]	0.752	1226	0.78 [0.21, 2.9]	0.72	3610	1.03 [0.2, 5.35]	0.97	3610

^a^Complete case analyses include only participants who had data for all variables of the fully adjusted statistical models, maximum available data analyses include participants provided they had complete data for particular analysis (adjusted or unadjusted), imputed data analyses include participants who had complete data for unadjusted analyses (missing covariate data was imputed in the adjusted analyses)

^b^Adjusted models include the covariates: sex; socio-economic status; mother parity; mother age at delivery; mother lifetime AN; mother smoking; age 10 BMI, worry; baseline (age 14) value of outcome

^c^Adjusted models include the covariates: sex; socio-economic status; mother parity; mother age at delivery; mother lifetime AN; age 10 BMI, worry; baseline (age 14) value of outcome. Mother smoking was not included due to perfect prediction

##### Cross sectional prediction of smoking by anorexia nervosa

In unadjusted analyses AN was associated with greater heaviness of smoking at the same time-point, with strong statistical evidence to support the association (see Table 3). The strength of association reduced in adjusted analyses, though the statistical evidence for an association remained moderate. There was no evidence for a cross-sectional association between AN and smoking initiation in unadjusted or adjusted analyses.

**Table 3:** Results of cross-sectional analyses

	Association between Smoking Heaviness and anorexia nervosa					
Data^a^	Unadjusted			Adjusted^b^		
	OR [95% CI]	*P*	*N*	OR [95% CI]	*P*	*N*
Complete case	2.37 [1.27, 4.41]	0.01	3281	2.00 [1.07, 3.72]	0.03	3509
Maximum available	1.92 [1.11, 3.32]	0.02	5044	2.00 [1.07, 3.72]	0.03	3509
Imputed	1.92 [1.11, 3.32]	0.02	5044	1.52 [0.86, 2.69]	0.15	5044

	Association between Smoking Initiation and anorexia nervosa					
	Unadjusted			Adjusted^b^		
	OR [95% CI]	*P*		OR [95% CI]	*P*	
Complete case	0.77 [0.13, 4.52]	0.77	2982	0.96 [0.24, 3.82]	0.95	2982
Maximum available	0.62 [0.11, 3.68]	0.60	3807	0.96 [0.24, 3.82]	0.95	2982
Imputed	0.62 [0.11, 3.68]	0.60	3807	0.80 [0.22, 2.96]	0.74	3807

^a^Complete case analyses include only participants who had data for all variables of the fully adjusted statistical models, maximum available data analyses include participants provided they had complete data for particular analysis (adjusted or unadjusted), imputed data analyses include participants who had complete data for unadjusted analyses (missing covariate data was imputed in the adjusted analyses)

^b^Adjusted models include the covariates: sex; socio-economic status; mother parity; mother age at delivery; mother lifetime AN; mother smoking; age 10 BMI, worry; baseline (age 14) value of outcome

##### Complete case and maximum available data analyses

Point estimates of associations in complete case and maximum available data analyses were generally consistent with the pattern of results from imputed data analyses (see Tables 2 and 3). The statistical evidence in respect of all associations was weak across these analyses, with two exceptions. In adjusted analyses there was moderate support for greater heaviness of smoking predicting increased risk of subsequent AN, and for positive cross-sectional associations between AN and smoking heaviness. Full results are displayed in Tables 2 and 3.

#### Study two

##### The effect of anorexia nervosa on smoking behaviour

**Fig. 2:**
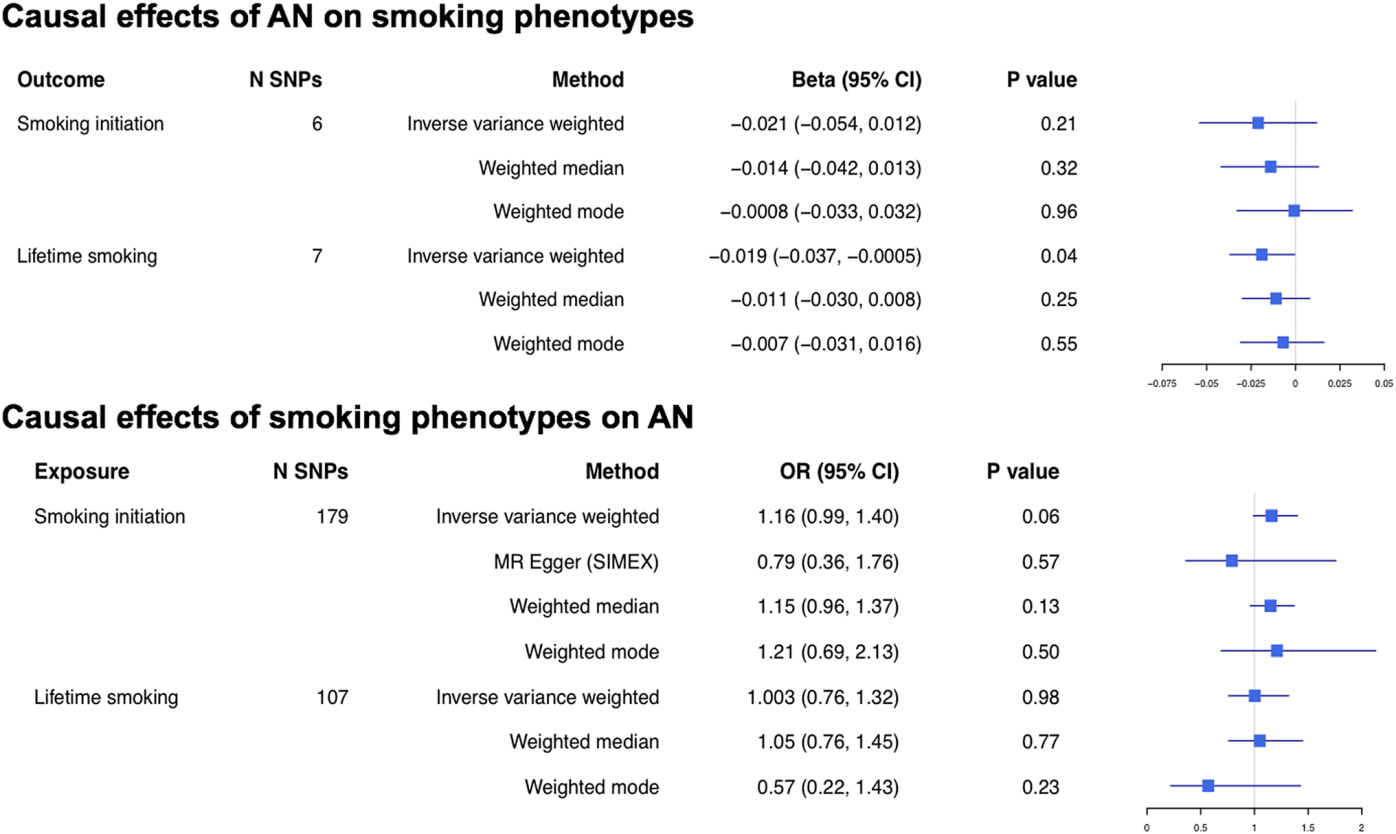
Two-sample MR exploring the bi-directional effects between AN and smoking behaviour. When smoking initiation was the instrument, MR Egger SIMEX correction was conducted to adjust for intermediate $I_{{{\text{GX}}}}^{2}$ (see Supplement, Table S5). When AN and lifetime smoking were the exposures, neither MR Egger or MR Egger SIMEX could not be conducted due to low $I_{{{\text{GX}}}}^{2}$

In analyses using a more stringent threshold to identify instruments (*p* < 5 × 10^–8^), there was no clear evidence for an effect of AN on smoking initiation or lifetime smoking, with inconsistent effect estimates across the methods and wide confidence intervals (Fig. 2, Supplementary Figs. S2–3). Outcomes of sensitivity analyses using a lower threshold for instrument identification (*p* < 5 × 10^–5^) also provided no clear evidence for causal effects (Supplement, Table S6), with *F* statistics indicating no weak instruments (Supplement, Table S7). There was evidence of heterogeneity in the SNP effects (Supplement, Table S8). MR Egger intercepts did not suggest bias from directional pleiotropy (Supplement, Table S9); however, the estimates should be considered with caution due to low $I_{{{\text{GX}}}}^{2}$. There was no evidence of outlier SNP effects (Supplement, Figs. S4–7).

##### The effect of smoking behaviour on anorexia nervosa

There was no clear evidence for an effect of either smoking behaviour on risk of AN and effects were inconsistent across all estimation methods (Fig. 2, Supplementary Figs. S8–9). SNP effects were heterogeneous (see Supplement, Table S8), but there was no evidence from the MR Egger intercept that bias from directional pleiotropy affected the IVW estimates (see Supplement, Table S9). The MR Egger indices of pleiotropy should be interpreted cautiously for the lifetime smoking exposure given low $I_{{{\text{GX}}}}^{2}$. However, SIMEX corrections were possible for MR Egger analyses assessing effects of smoking initiation (see Table S7). There was no evidence of outlier SNP effects (Supplement, Figs. S10–13). A full list of SNPs used in all MR analyses are given in Supplementary Tables S10–S13.

## Discussion

This research explored the association between smoking behaviour and AN. Findings were triangulated across two study designs (longitudinal observational, and MR), which were subject to different sources of bias, to improve the accuracy of inferences. It was hypothesised that associations consistent with bi-directional causal effects between smoking and AN would be observed. Contrary to hypotheses, we found no clear evidence to support longitudinal associations between AN and smoking behaviour. Outcomes of MR analyses were consistent, producing no evidence to support causal associations between AN and smoking.

Prospective associations between smoking behaviour and AN have not to our knowledge been explored previously. The absence of association of AN with either smoking initiation or heaviness is novel and informative, though does conflict with prior reports that elevated AN psychopathology (i.e. weight concern) is associated with increased risk of smoking in adolescent populations [11, 50]. The discrepancy may be explained by AN diagnosis and AN psychopathology being different phenotypes, with a population high in weight concern being more diverse, and likely less harm avoidant, than individuals with a diagnosis of AN. The lack of evidence for a cross-sectional association between AN and smoking contrasts with previous case–control studies that have reported an increased prevalence of smoking [8] or nicotine dependence [7] in AN relative to HC. Differences in sample demographic characteristics between studies could account for differing findings. The age of our sample was younger than that of studies previously described, and it is possible associations strengthen in adulthood. Indeed smoking prevalence is more common amongst adults with AN as compared to adolescents with AN [51]. Alternatively, the fact we adjusted for various confounders in the analysis, while statistical control in prior studies has been somewhat limited [51], may explain conflicting findings. Consistent with this explanation, there was evidence to support cross-sectional associations between AN and smoking heaviness in unadjusted data analyses. The associations identified in prior studies may reflect the existence of shared risk factors for AN and smoking that were not accounted for in the analysis.

In Study 2, there was also no clear evidence for a causal effect of AN on smoking behaviour, or of causal effects of smoking behaviour on AN. Given the MR design of Study Two reduces bias from confounding, this outcome supports the proposal that previously reported cross-sectional associations between AN and smoking (e.g. [7, 8]) may to some extent be explained by operation of a third unmeasured factor. One plausible shared risk factor is negative affect, comprising anxious and depressed mood, for which the worry we adjusted for in multivariable observational analyses may serve as a proxy [52]. Negative affect has been associated with subsequent initiation of smoking and smoking heaviness [53], as well as with AN [54, 55]. Evidence supports causal effects of negative affect on smoking in smokers (e.g. [56–58] and on disordered eating in AN (e.g. [59, 60]). Furthermore, smokers report reductions in negative affect following smoking [61, 62] and individuals with AN report reductions in negative affect following engagement in disordered eating [60]. Smoking and restrictive eating may thus reflect an underlying proneness to negative affect, with both behaviours functioning as maladaptive coping strategies.

The existence of shared risk factors might be expected to play out in longitudinal as well as cross-sectional associations. However, effects of such risk factors could be proximal, and levels of the risk factors dynamic over time. In addition, shared risk factors of smoking and AN are no doubt common to a number of other health risk behaviours and psychiatric disorders [63–66]. An underlying vulnerability may be expressed in various different ways, which may change over time with the developmental context. This would further increase the dependency of detecting associations between smoking and AN on the timing of assessment. Targeting those factors that causally increase risk for both substance use and disordered eating in prevention efforts (e.g. [67]) comprises a cost-effective strategy for improving public health. A priority area for future research is to confirm potential shared causal risk factors as such, and to develop novel prevention interventions accordingly. Notably, this strategy is likely to be more useful when considering a wider collection of health behaviours and psychiatric symptoms.

In each of the studies we took several measures to increase the accuracy of effect estimates and conclusions. In Study One, associations were assessed across multiple waves of data, and missing covariate data were imputed, to maximise use of available AN and smoking variable observations. Analyses were conducted with imputed, maximally available and complete case data. Point estimates and corresponding inferences were generally consistent across analyses, strengthening confidence in conclusions. We adjusted our analyses to account for effects of potential confounding factors, took the clustered nature of repeated measures data into account and considered mechanisms surrounding data missingness, to promote unbiased inferences. The large sample size increases reliability and validity of resulting conclusions. In Study Two, we used a Mendelian randomisation design, which reduces bias from confounding and reverse causation, to strengthen causal inference [26]. The MR method is particularly powerful for phenotypes such as smoking and AN diagnosis, which are either impossible or unethical to randomise, meaning effects of these exposures cannot be investigated using a randomised control trial. Confounding can be reintroduced via pleiotropic pathways. We conducted multiple sensitivity analyses to determine bias arising from pleiotropy and judged the strength of evidence generated from the MR analyses by looking for consistent effect estimates across multiple methods. Generally, effect estimates were close to zero, or confidence intervals were wide, enhancing confidence in the conclusion that there is no strong statistical evidence for an effect in either direction.

Each of the two studies had limitations. In Study One, AN diagnoses were assigned using a population assessment strategy, rather than resulting from clinical interviews or clinician evaluations. Measurement error may have acted to dampen associations when AN was the exposure and reduce statistical power when AN was the outcome [68]. Residual confounding is probable, given it is unlikely all confounders were sufficiently accounted for in the analysis [69]. The number of AN cases was low in the prospective investigation (Study One), rendering analyses with the AN outcome vulnerable to rare events bias, and analyses with the AN exposure vulnerable to data sparsity effects, particularly in adjusted analyses. AN disproportionately affects females, potentially increasing issues with data sparsity when covarying for gender in our analyses. Reassuringly, however, coefficients and standard errors of the observational analyses did not show signs of the effect size inflation that is symptomatic of rare events/data sparsity bias.

Study Two is not vulnerable to these sources of bias;, however, alternative causes of error are plausible. The largest source of bias in MR studies is pleiotropic effects. Though we used MR Egger to formally quantify such effects (finding no indication of bias due to pleiotropy), these estimates should be interpreted with caution due to variance of the SNP-exposure associations [43]. We did not have an AN sample stratified by smoking status and, therefore, were unable to assess distinct effects between AN and smoking duration/heaviness/cessation, which may have prevented us from detecting true associations in MR analyses. However, the lifetime smoking measure indexes smoking heaviness in unstratified samples, and results of analyses using this measure showed no evidence for causal effects. MR analyses with the AN exposure are likely to be very underpowered, since only 7 genome-wide significant SNPs were available for use as AN instruments, and power in MR analyses is primarily dictated by the variance in exposure explained by the SNP instruments [70]. Though a sensitivity analysis with a relaxed *p*-value threshold for instrument identification still resulted in no clear evidence for causal effects, we note that estimates will be biased towards the null when using weaker instruments (less strongly associated with the exposure) [71]. It is also important to note that MR estimates lifetime effects, and, therefore, is not sensitive to particular critical windows or developmental stages. However, this makes MR complementary to longitudinal observational analyses focusing on particular life stages, as in Study One, in which we examined associations between AN and smoking during the adolescent period. An assumption of the two-sample MR method is that the exposure and outcome GWAS are conducted in homogenous populations, with heterogeneity leading to possible bias [72]. The GWAS included individuals with European ancestry only; however, there was a large sex discrepancy, with around 54% of participants in both smoking GWAS being female, compared to an estimated 97% of participants in the AN GWAS. Genetic variants for AN previously identified in a female-only GWAS did predict AN risk in an all male sample [31], suggesting that these variants should still be valid instruments for AN in a smoking GWAS of mixed sex. Finally, two-sample MR can be biased by sample overlap in the exposure and outcome samples [73]. UK Biobank was included in GSCAN, and the population in which the composite smoking instrument was constructed, and UK Biobank also provided 768 cases and 3065 controls to the AN GWAS. Sample overlap is unlikely to have affected the interpretation of our results given it was small. Furthermore, such overlap would bias results towards the confounded observational estimate (and away from the null; [73]), yet there was no evidence for an association across analyses.

As the sources of bias differed across studies, the consistent results across the two studies allow for greater confidence that AN and smoking are not causally related, as compared to inferences based on outcomes of only one of the studies. However, it is possible for biases to have acted in the same way across the two studies (i.e. towards the null), which would undermine the strength of triangulating evidence across investigations of differing design. Across both studies we were not able to probe differential associations of smoking behaviour between AN subtypes. In the observational study this would have resulted in data sparsity and low power given the small number of AN cases. Use of summary GWAS data in MR analyses did not allow for subtype identification and separate group analyses either, though it has been reported that the genetic basis of AN does not vary by subtype [31]—consistent with the high level of cross over from restrictive to binge-purge AN [74]. However, there is some evidence to suggest prior associations identified between AN and smoking are driven by greater smoking in binge-purge AN, with reduced likelihood of smoking in restrictive AN [7, 75, 76]. Effect modification by subtype could therefore have prevented detection of true causal effects in both studies. MR analyses with the smoking initiation exposure, and prospective analyses of the association between smoking initiation and subsequent AN development, lacked power to detect small effects (these analyses had less than 80% power to detect an OR below 1.45 and 1.8, respectively), which could equally result in outcomes of Studies One and Two leading to the same wrong conclusion. True effects may well be smaller than expectations arising from prior cross-sectional findings, given confounding in cross-sectional studies is likely. As AN psychopathology in nonclinical populations is associated with a greater likelihood of smoking (e.g., [50, 75]), the effects of AN per se on smoking risk may be particularly small.

## Conclusion

Across two complementary studies, each with particular strengths, limitations and sources of bias, there was no evidence for causal effects between AN and smoking. In unadjusted analyses, AN was cross-sectionally associated with greater heaviness of smoking, potentially indicating the existence of shared risk factors for smoking and AN pathology. Elucidating such shared risk factors may highlight novel targets of prevention efforts able to simultaneously address health risk behaviour and psychiatric symptoms, to reduce the overall burden of ill-health in the population. Low power and effect modification by AN subtype could have masked meaningful relationships in the current study and should be considered in future investigations.

## Supplementary Information

Below is the link to the electronic supplementary material.

Supplementary file1 (DOCX 2952 kb)

Supplementary file2 (XLSX 53 kb)

## Data Availability

GWAS summary statistics are publicly available.
