# Supplementary material for: The absence of association between anorexia nervosa and smoking: converging evidence across two studies
Source: Eur Child Adolesc Psychiatry. 2021 Dec 22;32(7):1229–40. doi: 10.1007/s00787-021-01918-z (PMC10276073; doi:10.1007/s00787-021-01918-z)
Supplement: Supplementary file 1 — Supplementary file1 (DOCX 2952 kb) [file 787_2021_1918_MOESM1_ESM.docx]

**Supplementary Materials**

Contents

| **Tables** |  |
| --- | --- |
| Table S1. Measurement of Smoking behaviour in the ALSPAC cohort | Page 2 |
| Table S2. Measurement of AN symptoms in the ALSPAC cohort | Page 5 |
| Table S3. Comparison of Unimputed and Imputed Covariate Data | Page 7 |
| Table S4. Mendelian randomisation power calculation | Page 8 |
| Table S5. ALSPAC Data Collection and Demographic Characteristics of Participants | Page 9 |
| Table S6. Sensitivity MR analysis with a relaxed p-value threshold for the AN Instrument | Page 12 |
| Table S7. Instrument suitability for the MR Egger test | Page 13 |
| Table S8. Cochran’s Q test for SNP-effect heterogeneity | Page 14 |
| Table S9. MR Egger intercept test for bias from directional pleiotropy | Page 15 |
| Tables S10-13 are available in a separate supplementary Excel file.  **Figures** |  |
| Figure S1 Collection and availability of smoking and AN data in the ALSPAC cohort | Page 16 |
| Figures S2-S3. Scatter plots of SNP-AN on SNP-smoking effects | Page 17 |
| Figures S4-S5. Single SNP Wald ratio estimates for the effect of AN on smoking | Page 19 |
| Figures S6-S7. Leave-one-out analyses for the effect of AN on smoking | Page 21 |
| Figures S8-S9. Scatter plots of SNP-smoking on SNP-AN effects | Page 23 |
| Figures S10-S11. Single SNP Wald ratio estimates for the effect of smoking on AN | Page 25 |
| Figures S12-S13. Leave-one-out analyses for the effect of smoking on AN | Page 27 |
| **References** | Page 29 |

**Measurement of Smoking Behaviour in the ALSPAC Cohort**

*Table S1 Smoking Variable Derivation*

| Age  Source | 13 years (N=5813)  Computer in clinic | 14 years (N=5671)  Postal questionnaire | 15 years (N=5107)  Computer in clinic | 16 years (N=4853)  Postal questionnaire | 17 years (N=3954)  Computer in clinic | 18 years (N=3209)  Postal questionnaire | 20 years  Postal questionnaire |
| --- | --- | --- | --- | --- | --- | --- | --- |
| Non-smoker | “ever smoked a cigarette”= “No”  OR if “still smokes” = “No”  OR if “smoking habits in previous 6 months” = “no smoking” | “ever smoked a cigarette”= “No”  OR if “current smoking behaviour”  = “I used to smoke sometimes but I never smoke cigarettes now”  OR if “current smoking behaviour”  =”I have only ever tried smoking cigarettes once or twice” | “tried a cigarette”=”No”  OR “smoked in last 30 days” = “No” | “ever smoked a cigarette”= “No”  OR if “current smoking behaviour”  = “I used to smoke sometimes but I never smoke cigarettes now”  OR if “current smoking behaviour”  =”I have only ever tried smoking cigarettes once or twice” | “smoked a whole cigarette”=”No”  OR “smoked in last 30 days” = “No” | “smoked a whole cigarette”=”No”  OR “smoked in last 30 days” = “No” | “smoked a whole cigarette”=”No”  OR “smoked in last 30 days” = “No” |
| Occasional smoker | “ever smoked a cigarette”= “Yes”  AND if “still smokes” = “Yes”  AND if “smoking habits in previous 6 months” = “1-3 times per month” OR “more than 4 times” | “ever smoked a cigarette”= “Yes”  AND if “current smoking behaviour”  =”I sometimes smoke cigarettes but I smoke less than one a week” | “tried a cigarette”=”Yes”  AND “smoked in last 30 days” = “Yes”  AND “smokes weekly” = “No”  AND “smokes daily”=”No” | “ever smoked a cigarette”= “Yes”  AND if “current smoking behaviour”  =”I sometimes smoke cigarettes but I smoke less than one a week” | “smoked a whole cigarette”=”Yes”  AND “smoked in last 30 days” = “Yes”  AND “smokes weekly” = “No”  AND “smokes daily”=”No” | “smoked a whole cigarette”=”Yes”  AND “smoked in last 30 days” = “Yes”  AND “smokes weekly” = “No”  AND “smokes daily”=”No” | “smoked a whole cigarette”=”Yes”  AND “smoked in last 30 days” = “Yes”  AND “smokes weekly” = “No”  AND “smokes daily”=”No” |
| Weekly smoker | “ever smoked a cigarette”= “Yes”  AND if “still smokes” = “Yes”    AND if “smoking habits in previous 6 months” = “once per week” | “ever smoked a cigarette”= “Yes”  AND if “current smoking behaviour”  =”I usually smoke between one and six cigarettes a week”  OR “I usually smoke more than six cigarettes a week, but not every day” | “tried a cigarette”=”Yes”  AND “smoked in last 30 days” = “Yes”  AND “smokes weekly” = “Yes” | “ever smoked a cigarette”= “Yes”  AND if “current smoking behaviour”  =”I usually smoke between one and six cigarettes a week”  OR “I usually smoke more than six cigarettes a week, but not every day” | “smoked a whole cigarette”=”Yes”  AND “smoked in last 30 days” = “Yes”  AND “smokes weekly” = “Yes” | “smoked a whole cigarette”=”Yes”  AND “smoked in last 30 days” = “Yes”  AND “smokes weekly” = “Yes” | “smoked a whole cigarette”=”Yes”  AND “smoked in last 30 days” = “Yes”  AND “smokes weekly” = “Yes” |
| Daily smoker | N/A ^a^ | “ever smoked a cigarette”= “Yes”  AND if “current smoking behaviour”  =”I usually smoke one or more cigarettes every day” | “tried a cigarette”=”Yes”  AND “smoked in last 30 days” = “Yes”  AND “smokes daily”=”Yes” | “ever smoked a cigarette”= “Yes”  AND if “current smoking behaviour”  =”I usually smoke one or more cigarettes every day” | “smoked a whole cigarette”=”Yes”  AND “smoked in last 30 days” = “Yes”  AND “smokes daily”=”Yes” | “smoked a whole cigarette”=”Yes”  AND “smoked in last 30 days” = “Yes”  AND “smokes daily”=”Yes” | “smoked a whole cigarette”=”Yes”  AND “smoked in last 30 days” = “Yes”  AND “smokes daily”=”Yes” |

^a^ There was no assessment of daily smoking at this time point

**Measurement of AN symptoms in the ALSPAC cohort**

*Table S2 Criteria Used to Derive Anorexia Nervosa Diagnoses*

| Age | Weight criteria | Child report | Parent report |
| --- | --- | --- | --- |
| 14 | Underweight | Weight/shape concern OR engaged in fasting for weight loss or to avoid weight gain at least monthly OR engaged in excessive exercise | Presence of fear of weight gain AND fat avoidance in the 3 months prior to assessment |
| 16 | Underweight | Engaged in fasting for weight loss or to avoid weight gain at least monthly OR engaged in excessive exercise | Presence of fear of weight gain AND fat avoidance in the 3 months prior to assessment |
| 18 | Underweight | Weight/shape concern OR engaged in fasting for weight loss or to avoid weight gain at least monthly OR engaged in excessive exercise | N/A |

Disordered eating and exercise behaviour were ascertained from responses to questions adapted from the Youth Risk Behaviour Surveillance System [1] that were administered to participants at each wave. Fasting was assessed with the question “How often in the past year have you fasted (not eaten for at least a day) to lose weight or avoid gaining weight?”. Excessive exercise was recorded when participants endorsed exercising for weight-loss or to avoid weight-gain *and* at least one of the following: exercising despite illness/injury; exercise interfering with other activities; experiencing guilt when missing an exercise session. Body dissatisfaction was assessed at waves 14 and 18, using questions of the weight/shape concern scale of the McKnight Risk Factor Questionnaire [2]. The number of items administered varied with wave; body dissatisfaction was considered present if the mean response met a previously used threshold that corresponded to high levels [3]. Parent reported child AN symptoms were collected using the Development and Wellbeing Assessment (DAWBA) assessment [4], and marked as present when these were severe or extreme. Objective height and weight measurements collected during clinic assessments at each of the waves was used to determine whether participants were underweight, using UK gender-specific norms. Underweight corresponded to WHO grade 1 thinness [5].

**Measurement of covariates**

Child worry was assessed using the parent-report DAWBA [4]. Parents were asked if their child worried, and subsequently whether children worried across seven domains: past behaviour; school work; disasters; health; bad things happening; the future; and other things. The variable was the sum of responses across worry content items, with possible options ‘not at all’ (coded 0), ‘sometimes’ (coded 1), and ‘often’ (coded 2). Worry was coded 0 if the response to the first question ‘Does your child worry’ was ‘no’.

Data in respect of all other covariates was obtained from questionnaire data, apart from the BMI at age 10 z-score variable, which was derived from clinic-assessed height and weight, child gender, and UK reference data [6].

**Comparison of Unimputed and Imputed Covariate Data**

*Table S3 Comparison of Unimputed and Imputed Covariate Data*

|  | | Unimputed data | | Imputed data |
| --- | --- | --- | --- | --- |
|  | | **Proportion of cases** | | |
| Parent Occupation^a^ | |  | |  |
| Manual | | 0.23 | | 0.29 |
| Non-manual | | 0.77 | | 0.71 |
| N | | 12196 | | 2678 |
| Mother parity |  | |  | |
| Primipari | | 0.45 | | 0.47 |
| Multipari | | 0.55 | | 0.53 |
| N | | 12915 | | 1959 |
| Mother AN | |  | |  |
| No | | 0.95 | | 0.94 |
| Yes | | 0.05 | | 0.06 |
| N | | 7753 | | 7121 |
| Mother smoking | |  | |  |
| No | | 0.80 | | 0.75 |
| Yes | | 0.20 | | 0.25 |
| N | | 11819 | | 3055 |
|  | | **Unimputed data** | | **Imputed data** |
|  | | **Mean (SE)** | | |
| Mother age at delivery | | 27.99 (0.04) | | 27.92 (0.25) |
| N | | 13962 | | 912 |
| Child BMI at age 10 (z-score) | | 0.32 (0.01) | | 0.36 (0.02) |
| N | | 7457 | | 7416 |

^a^ Parent occupation is a proxy indicator for socio-economic status, with manual and non-manual occupations coded according to 1991 Office of Population, Censuses and Surveys classification.

**Mendelian Randomisation Power Calculation**

Table S4. Mendelian randomisation power calculation

|  | r2 | Outcome Sample Size | Effect size we have 80% power to detect |
| --- | --- | --- | --- |
| Smoking initiation -> AN | 2.30% | 72,517 (23% cases) | OR = 1.17 |
| Lifetime smoking -> AN | 0.36% | 72,517 (23% cases) | OR = 1.45 |
| AN -> Smoking initiation | 1.7%* | 632,802 | Beta = 0.03 |
| AN -> Lifetime smoking | 1.7% * | 462,690 | Beta = 0.03 |

*1.7% is the variance explained by a polygenic score of AN, rather than genome-wide significant variants specifically.

Power calculations were conducted using the online power calculator for Mendelian randomisation (<https://shiny.cnsgenomics.com/mRnd/>; [7]). This is designed for one-sample MR, therefore, here we input outcome sample size.

**ALSPAC Data Collection and Demographic Characteristics of Participants**

*Table S5 Sample Characteristics*

| Demographic Variable | *N* (%) | |
| --- | --- | --- |
| *Sex* |  | |
| Male | 2231 (43.75) | |
| Female | 2869 (56.25) | |
| *Parent Occupation^a^* |  | |
| Manual | 679 (13.31) | |
| Non-manual | 4030 (79.02) | |
| *Ethnicity* |  | |
| Non-white | 181 (3.83) | |
| White | 4539 (96.17) | |
| *Mother Parity* |  | |
| Primipari | 2,334 (45·76) | |
| Multipari | 2,428 (47·61) | |
|  | Mean (SD) | |
| Mother age at delivery* | 29.40 (4.48) | |
| Exposure and outcome variables, by sex | Male  N (%) | Female  N (%) |
| Smoking at 10 |  |  |
| No | 1960 (> 99) | 2941 (> 99) |
| Yes | < 5 (< 1) | < 5 (< 1) |
| Smoking at 14 |  | |
| None | 1998 (96.94) | 2524 (93.62) |
| Experimental | 31 (1.5) | 93 (3.45) |
| Weekly | 10 (0.49) | 35 (1.3) |
| Daily | 22 (1.07) | 44 (1.63) |
| *Initiation (n who initiated/n in analysis)* | 37/1703 | 94/2132 |
| Smoking at 16 |  | |
| None | 1532 (86.46) | 1944 (78.04) |
| Experimental | 96 (5.42) | 214 (8.59) |
| Weekly | 45 (2.54) | 131 (5.26) |
| Daily | 99 (5.59) | 202 (8.11) |
| *Initiation (n who initiated/n in analysis)* | 113/438 | 245/790 |
| Smoking at 18 |  | |
| None | 784 (76.26) | 1338 (73.48) |
| Experimental | 113 (10.99) | 195 (10.71) |
| Weekly | 39 (3.79) | 96 (5.27) |
| Daily | 92 (8.95) | 192 (10.54) |
| *Initiation (n who initiated/n in analysis)* | 47/138 | 66/330 |
| Smoking at 20 |  | |
| None | 883 (70.70) | 1451 (72.91) |
| Experimental | 154 (12.33) | 201 (10.10) |
| Weekly | 61 (4.88) | 103 (5.18) |
| Daily | 151 (12.09) | 235 (11.80) |
| *Initiation (n who initiated/n in analysis)* | 7/70 | |
| AN at 14 |  | |
| No | 2162 (> 99) | 2708 (> 99) |
| Yes | <5 (< 1) | 9 (< 1) |
| AN at 16 |  | |
| No | 2099 (> 99) | 2441 (98.91) |
| Yes | <5 (< 1) | 27 (1.09) |
| AN at 18 |  | |
| No | 1589 (> 99) | 2122 (> 99) |
| Yes | 5 (< 1) | 17 (< 1) |

*observed n = 4881

^a^ Parent occupation is a proxy indicator for socio-economic status, with manual and non-manual occupations coded according to 1991 Office of Population, Censuses and Surveys classification.

**Sensitivity MR analysis with a relaxed p-value threshold for the AN instrument**

Table S6. Two-sample Mendelian randomisation of Anorexia nervosa effects on smoking behaviours using a relaxed p-value threshold (p<5x10^-5^)

| Outcome | Method | N SNPs | Beta (95% CI) | P Value |
| --- | --- | --- | --- | --- |
| Smoking initiation | IVW | 159 | 0.00 (-0.01, 0.01) | 0.68 |
|  | MR Egger | 159 | 0.03 (0.00, 0.04) | 0.08 |
|  | Weighted median | 159 | 0.00 (0.01, 0.01) | 0.68 |
|  | Weighted mode | 159 | -0.01 (-0.02, 0.01) | 0.38 |
| Lifetime Smoking | IVW | 159 | 0.00 (-0.01, 0.01) | 0.87 |
|  | Weighted median | 159 | 0.00 (-0.01, 0.01) | 0.93 |
|  | Weighted mode | 159 | 0.00 (-0.01, 0.02) | 0.72 |

**Instrument suitability for the MR Egger test**

Table S7. I^2^_GX_ test of regression dilution to assess the suitability of the MR Egger test

|  | N SNPs | I2GX^a^ | Mean F |
| --- | --- | --- | --- |
| Smoking initiation -> AN | 179 | 0.62 | 49.43 |
| Lifetime smoking -> AN | 107 | 0.47 | 44.15 |
| AN (p<5x10-8) -> Smoking initiation | 6 | 0 | 35.94 |
| AN (p<5x10-8) -> Lifetime smoking | 7 | 0 | 35.32 |
| AN (p<5x10-5) -> Smoking initiation | 159 | 0.85 | 20.09 |
| AN (p<5x10-5) -> Lifetime smoking | 159 | 0 | 20.37 |

^a^ weighted I^2^_GX_ using first order weights

The F-statistic is a test of instrument strength, to check for possible weak instrument bias. We used the two-sample approximation outlined in Bowden et al. [8], calculating the F-statistic for each SNP independently. We present the mean of the F-statistics; F >10 indicates that weak instrument bias is unlikely.

Table S8. Cochran’s Q test for heterogeneity

|  | Cochran’s Q^a^ | df | P-value^b^ |
| --- | --- | --- | --- |
| Smoking initiation -> AN | 371 | 177 | 1.02 x 10^-15^ |
| Lifetime smoking -> AN | 209 | 106 | 9.02 x 10^-09^ |
| AN (p<5x10^-8^) -> Smoking initiation | 18 | 5 | 0.003 |
| AN (p<5x10^-8^) -> Lifetime smoking | 12 | 6 | 0.07 |
| AN (p<5x10^-5^) -> Smoking initiation | 371 | 158 | 3.31 x 10^-19^ |
| AN (p<5x10^-5^) -> Lifetime smoking | 426 | 152 | 8.23 x 10^-28^ |

^a^ Cochran’s Q given for the Inverse-variance weighted method, ^b^ statistical evidence that there is heterogeneity in SNP effect estimates

Table S9. MR Egger intercept test for bias from directional pleiotropy

|  | N SNPs | Beta (SE) | P-Value |
| --- | --- | --- | --- |
| Smoking initiation -> AN | 179 | 0.00 (0.00) | 0.41 |
| Smoking initiation -> AN (SIMEX) | 179 | 0.01 (0.01) | 0.35 |
| Lifetime smoking -> AN | 107 | -0.00 (0.01) | 0.62 |
| AN (p<5x10^-8^) -> Smoking initiation | 6 | -0.00 (0.01) | 0.39 |
| AN (p<5x10^-8^) -> Lifetime smoking | 7 | 0.00 (0.00) | 0.36 |
| AN (p<5x10^-5^) -> Smoking initiation | 159 | 0.00 (0.00) | 0.09 |
| AN (p<5x10^-5^) -> Smoking initiation (SIMEX) | 159 | 0.00 (0.00) | 0.33 |
| AN (p<5x10^-5^) -> Lifetime smoking | 159 | 0.00 (0.00) | 0.44 |

***
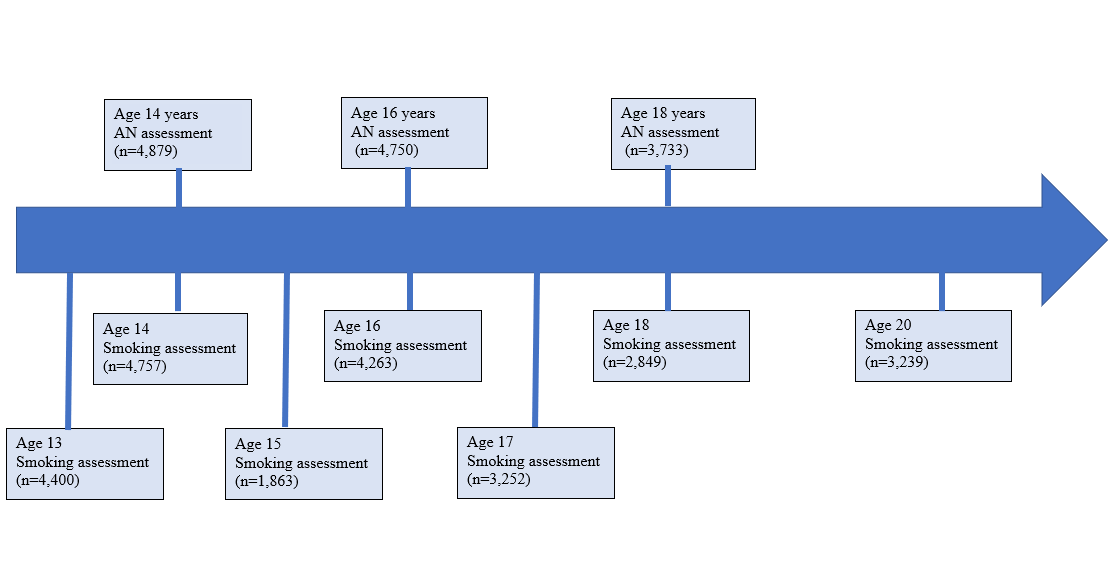
*  *Figure S1 Collection and availability of smoking and anorexia nervosa data in the ALSPAC cohort*  Plots for effects of AN on smoking behaviour**

Figure S2. Scatter plot of SNP-Exposure (AN) on SNP-Outcome (Smoking Initiation) Effects. Instrument-smoking initiation associations were regressed on instrument-AN associations using 4 MR methods (see legend). Y-axis scale represents per-allele log(odds ratio) of AN and X-axis scale represents standardised beta increase in the prevalence of smoking initiation.


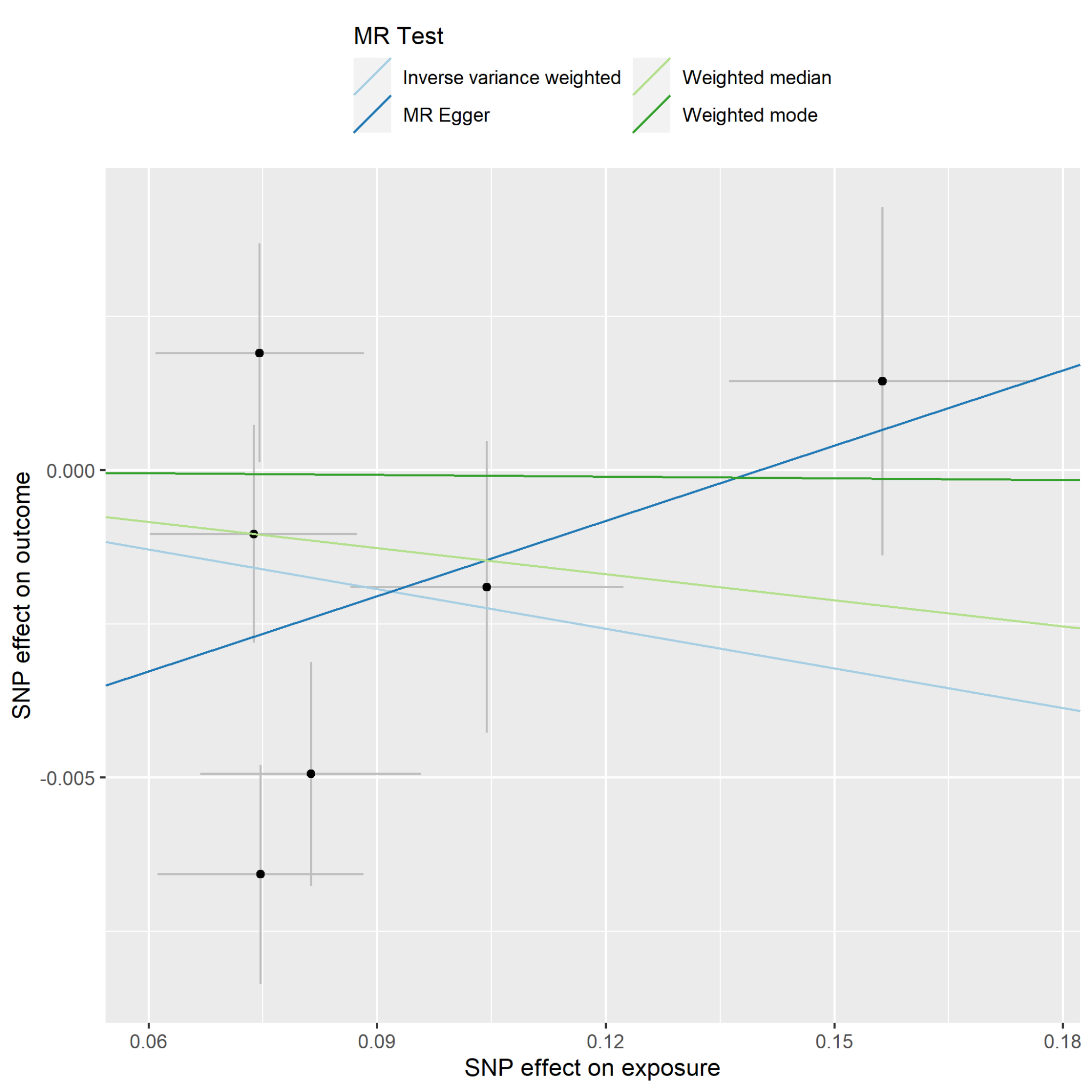


SNP effect on SI

SNP effect on AN

Figure S3. Scatter plot of SNP-Exposure (AN) on SNP-Outcome (Lifetime Smoking) Effects. Instrument-lifetime smoking associations were regressed on instrument-AN associations using 4 MR methods (see legend). Y-axis scale represents standardised beta increase in lifetime smoking index and X-axis scale represents per-allele log(odds ratio) of AN.


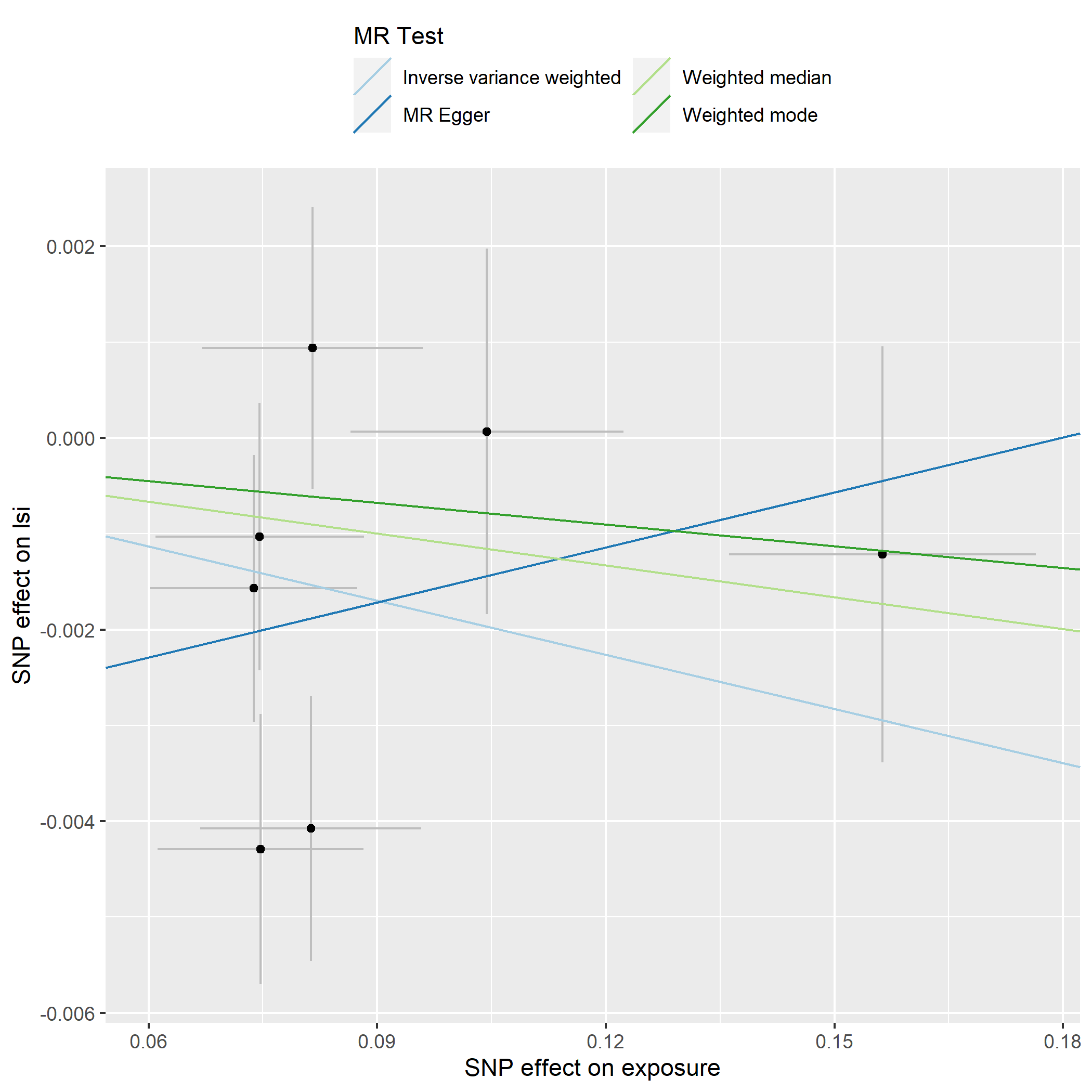


SNP effect on LSI

SNP effect on AN

Figure S4. Single SNP effects for Anorexia Nervosa on Smoking Initiation. Forest plot shows individual single nucleotide polymorphism (SNP) wald-ratio estimates (SNP-smoking initiation effect estimate / SNP-AN effect estimate). No outliers are identified as all single-SNP wald-ratios are consistent with the IVW main effect presented in red.


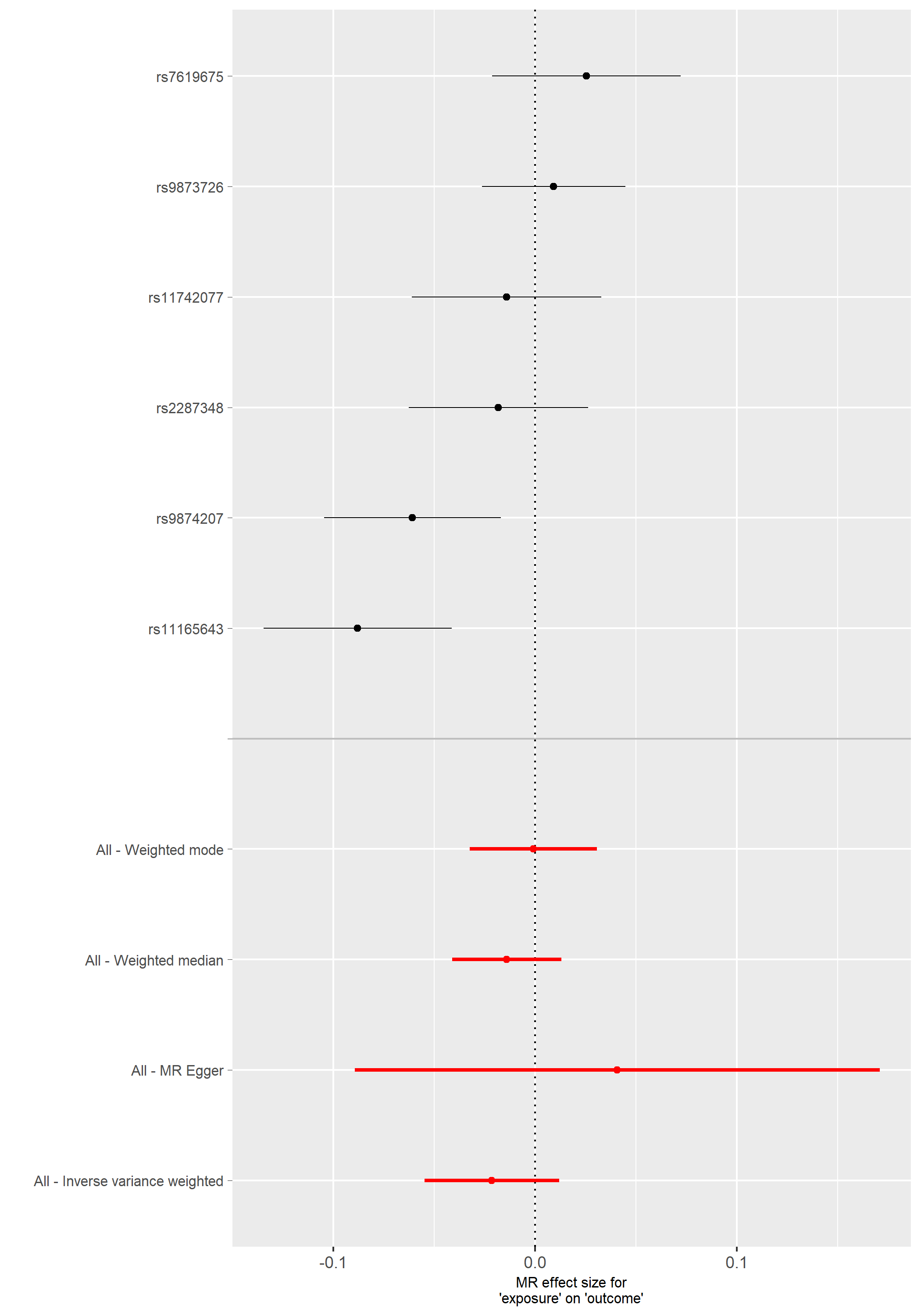


Figure S5. Single SNP effects for Anorexia Nervosa on Lifetime Smoking. Forest plot shows individual single nucleotide polymorphism (SNP) wald-ratio estimates (SNP-lifetime smoking effect estimate / SNP-AN effect estimate). No outliers are identified as all single-SNP wald-ratios are consistent with the IVW main effect presented in red.


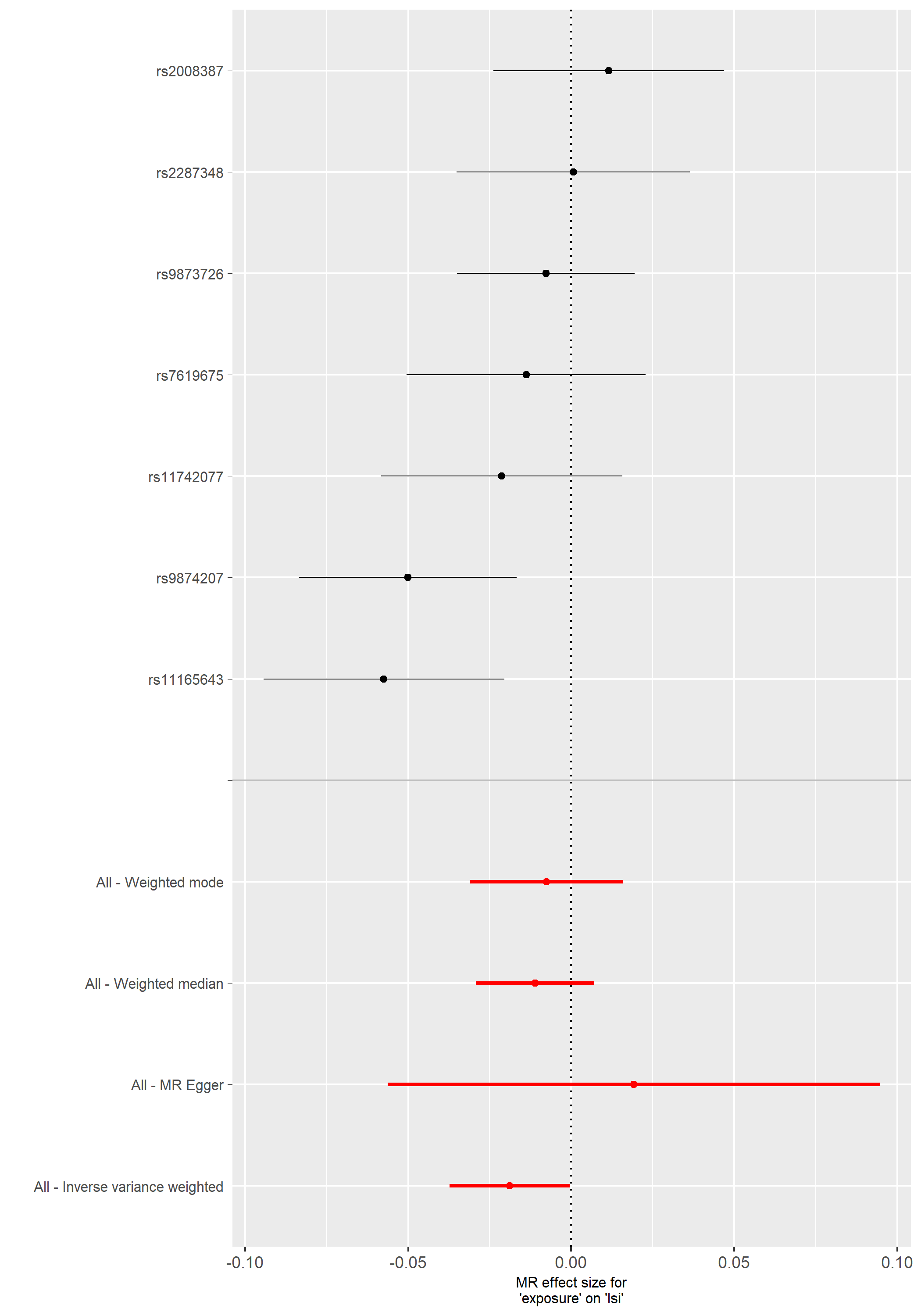


Figure S6. Leave-one-out sensitivity analysis for Anorexia Nervosa (p<5x10-8) on Smoking Initiation

Forest plot shows inverse variance weighted (IVW) estimates after omitting each SNP one at a time. No single-SNPs were driving the effects, as all of the leave-one-out analyses were consistent with the overall estimates in red.


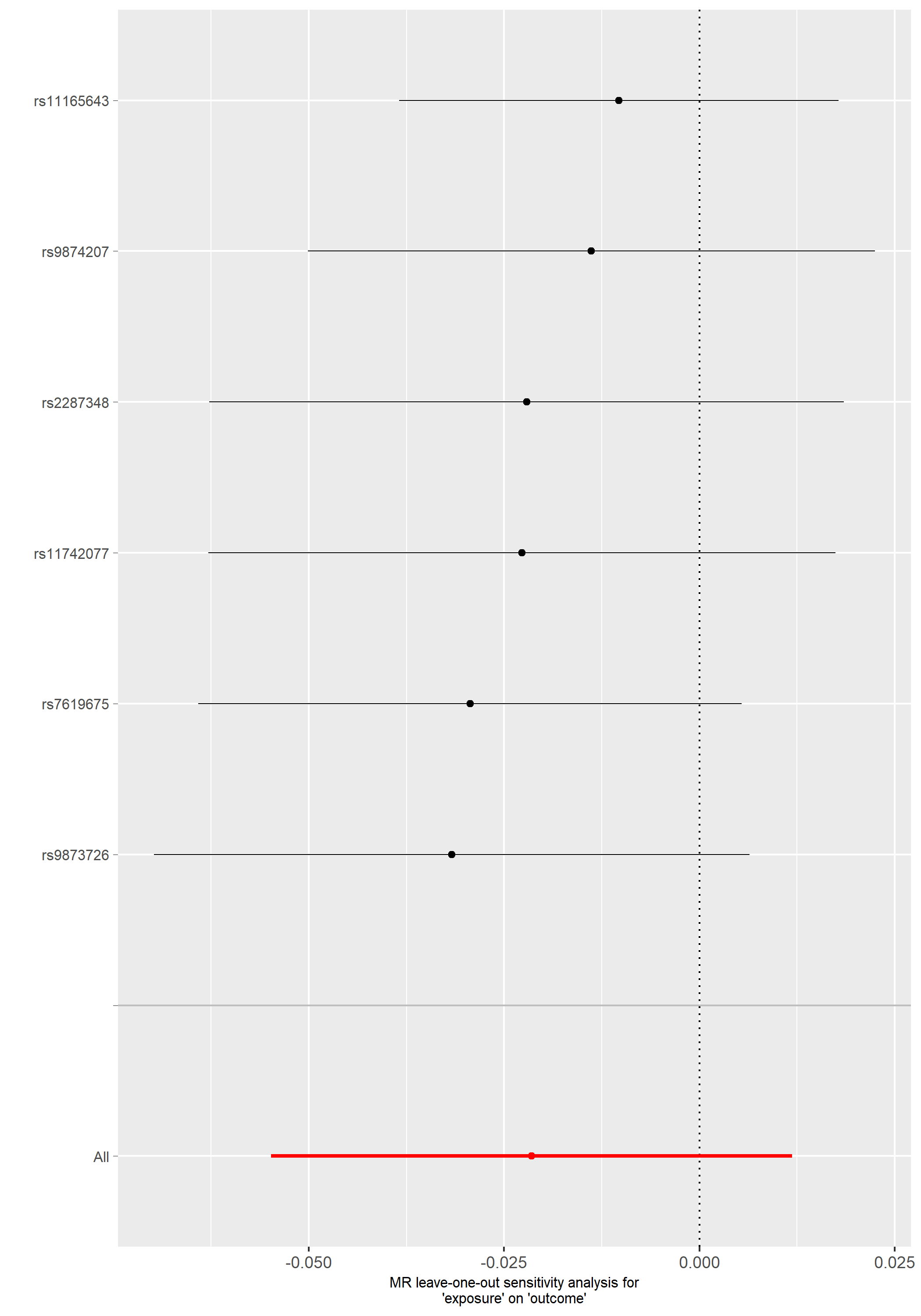


Figure S7. Leave-one-out sensitivity analysis for Anorexia Nervosa (p<5x10-8) on Lifetime Smoking. Forest plot shows inverse variance weighted (IVW) estimates after omitting each SNP one at a time. No single-SNPs were driving the effects, as all of the leave-one-out analyses were consistent with the overall estimates in red.


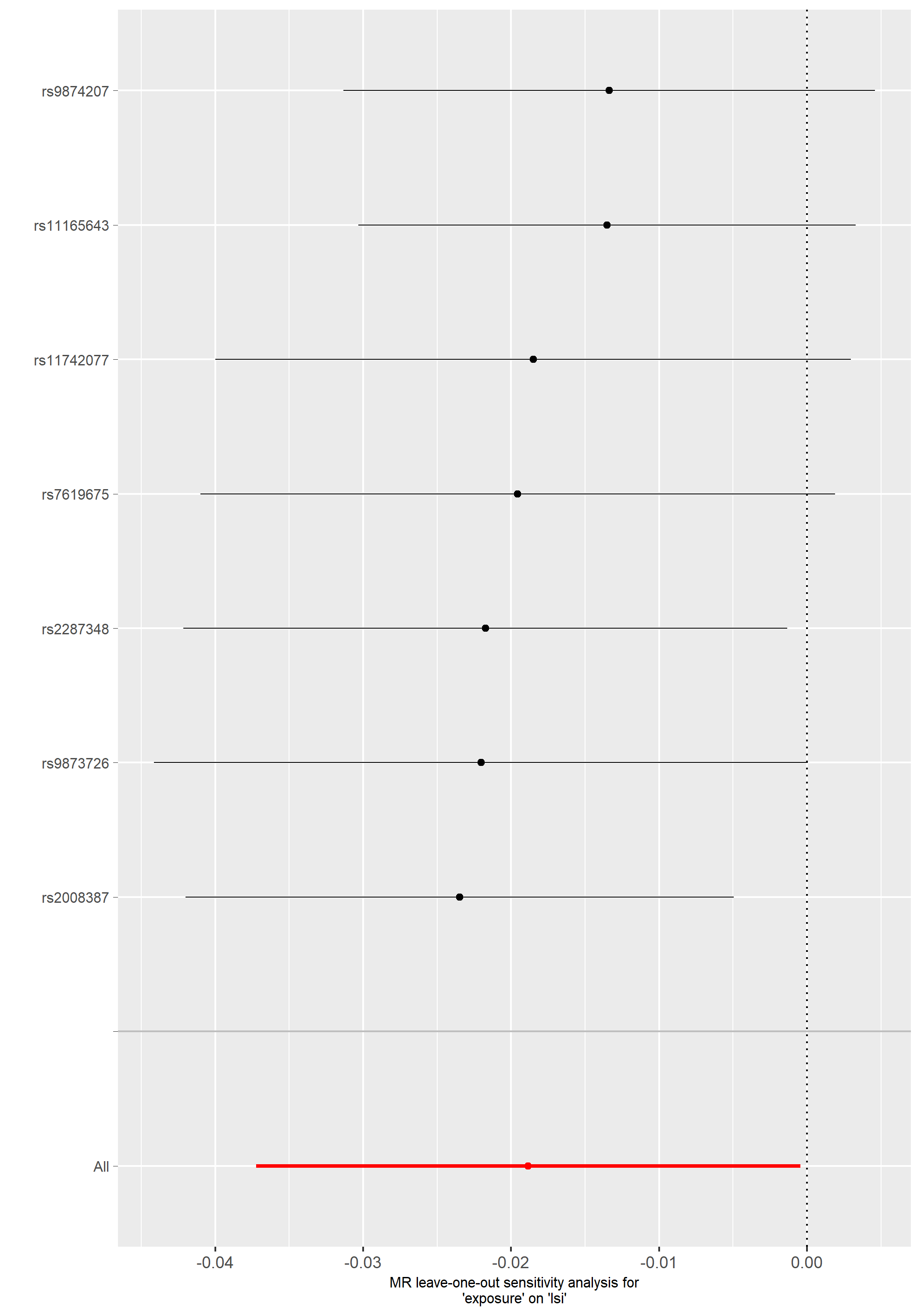


**Plots for effects of smoking behaviour on AN**

Figure S8. Scatter plot of SNP-Exposure (Smoking Initiation) on SNP-Outcome (AN) Effects. Instrument-AN associations were regressed on instrument-smoking initiation associations using 4 MR methods (see legend). Y-axis scale represents per-allele log(odds ratio) of AN and X-axis scale represents standardised beta increase in the prevalence of smoking initiation.


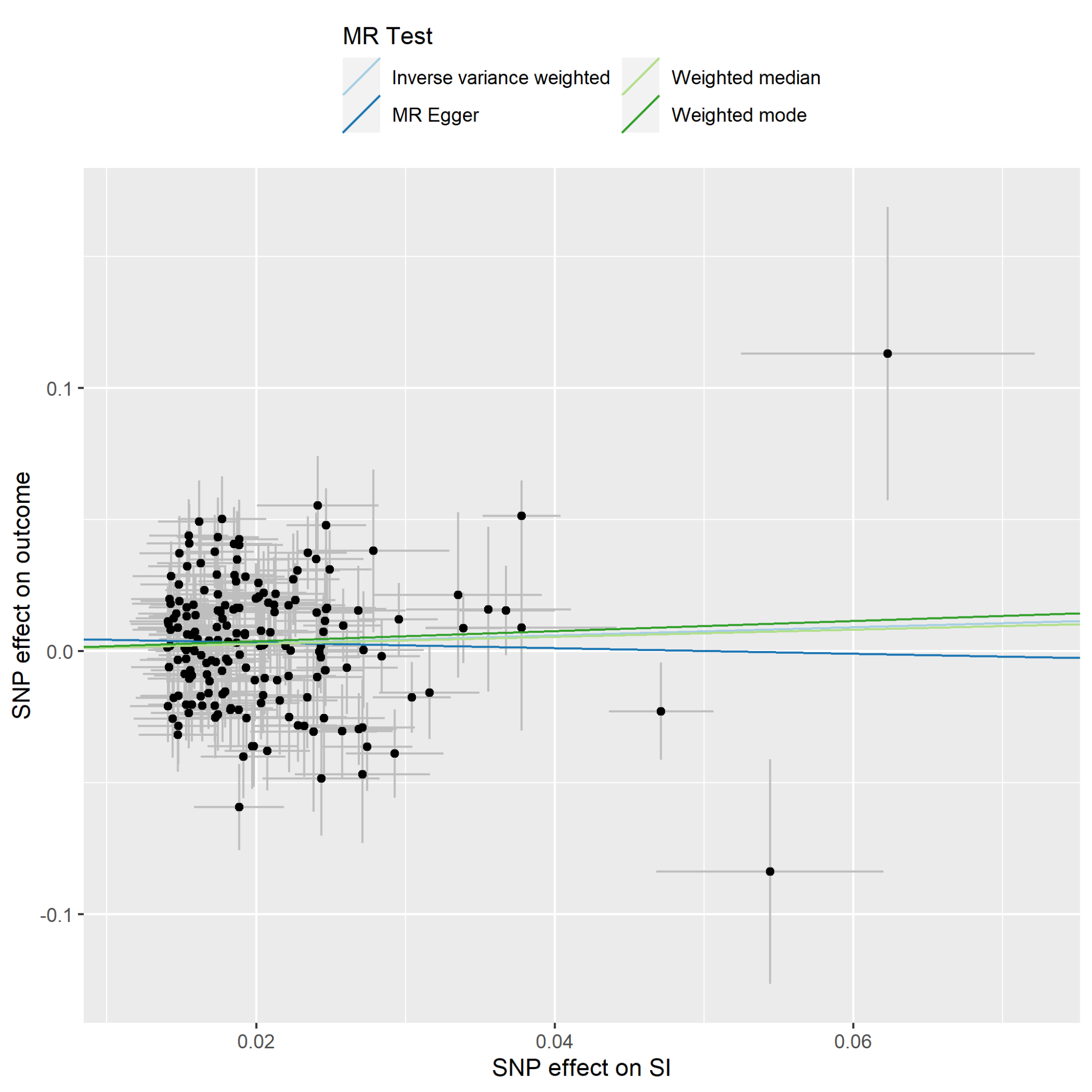


SNP effect on AN

Figure S9. Scatter plot of SNP-Exposure (Lifetime Smoking) on SNP-Outcome (AN) Effects. Instrument-AN associations were regressed on instrument-lifetime smoking associations using 4 MR methods (see legend). Y-axis scale represents per-allele log(odds ratio) of AN and X-axis scale represents standardised beta increase in lifetime smoking index.


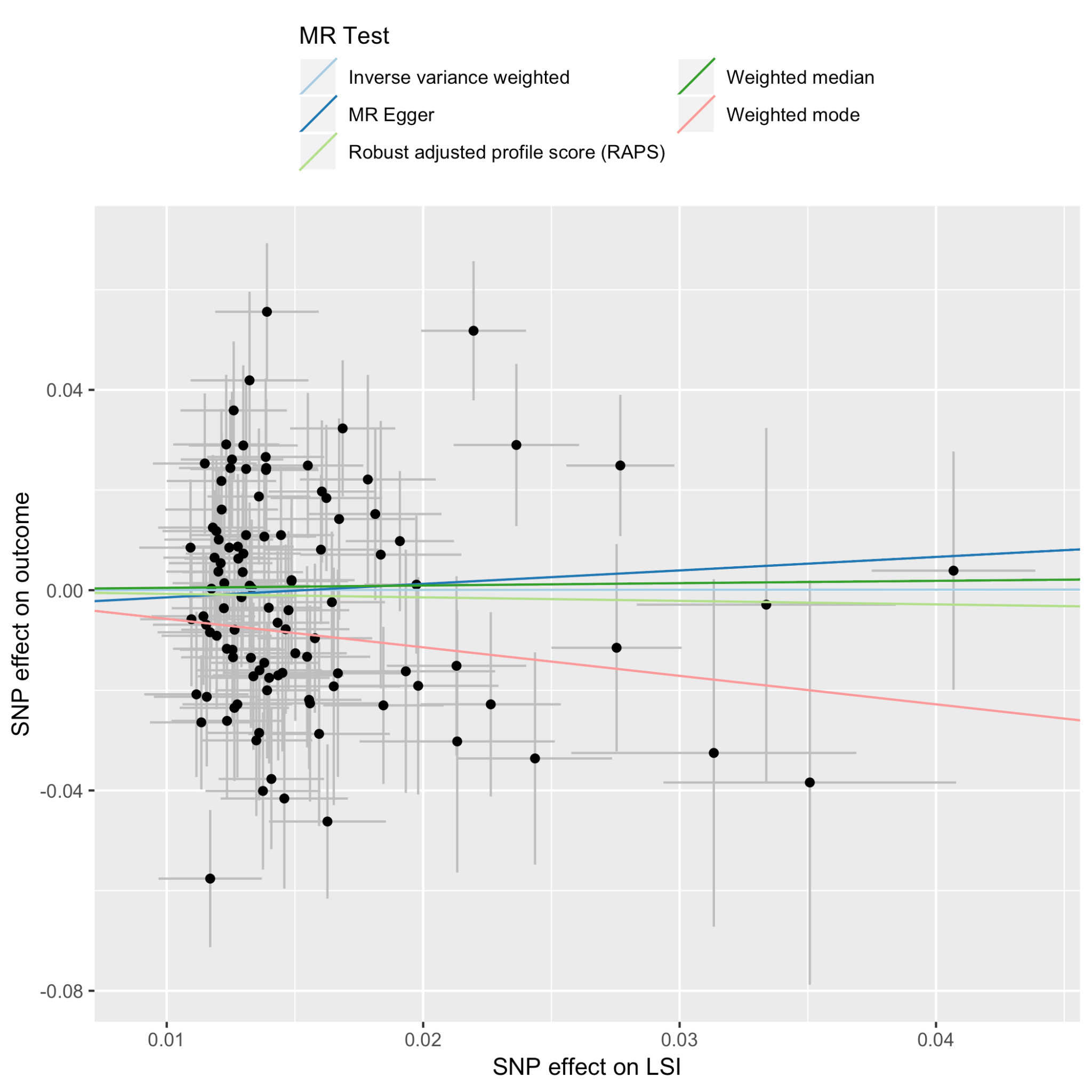


SNP effect on AN

Figure S10. Single SNP effects for Smoking Initiation on Anorexia Nervosa. Forest plot showing individual single nucleotide polymorphism (SNP) wald-ratio estimates (SNP-AN effect estimate / SNP-smoking initiation effect estimate). No outliers are identified as all single-SNP wald-ratios are consistent with the IVW main effect presented in red.


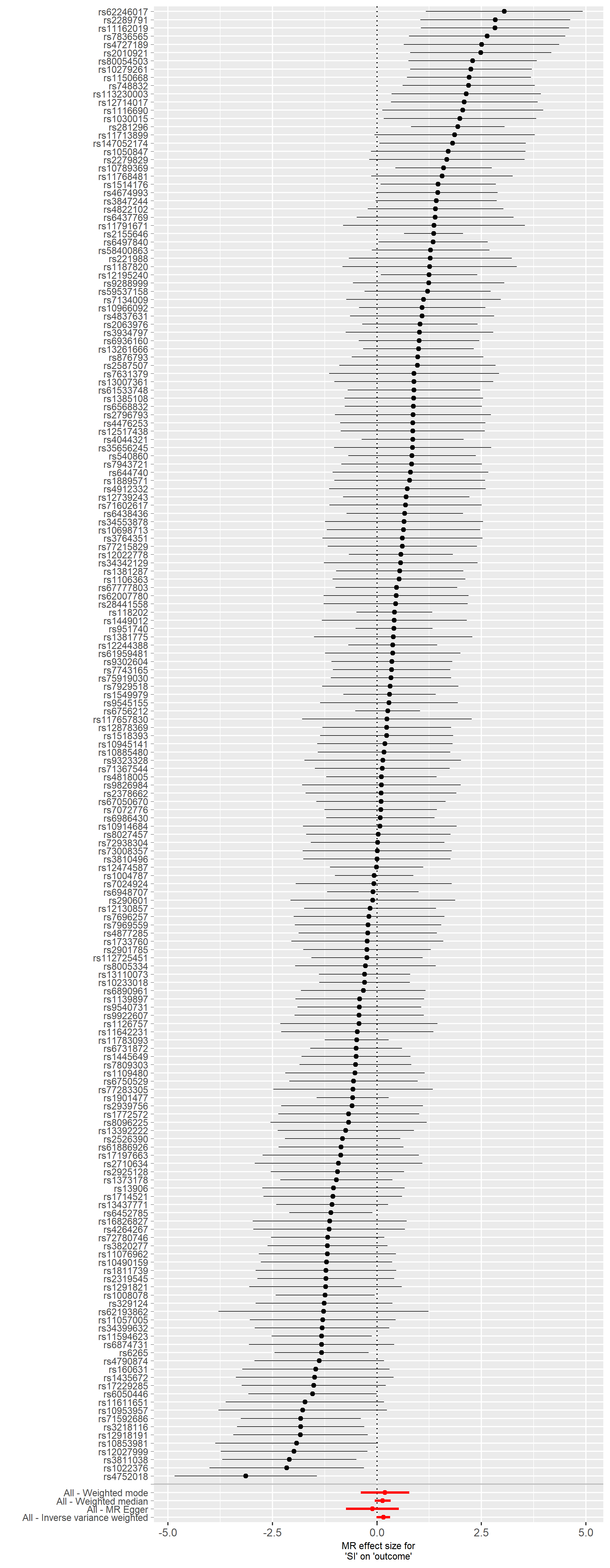


Figure S11. Single SNP effects for Lifetime Smoking on Anorexia Nervosa

Forest plot showing individual single nucleotide polymorphism (SNP) wald-ratio estimates (SNP-AN effect estimate / SNP-lifetime smoking effect estimate). No outliers are identified as all single-SNP wald-ratios are consistent with the IVW main effect presented in red.


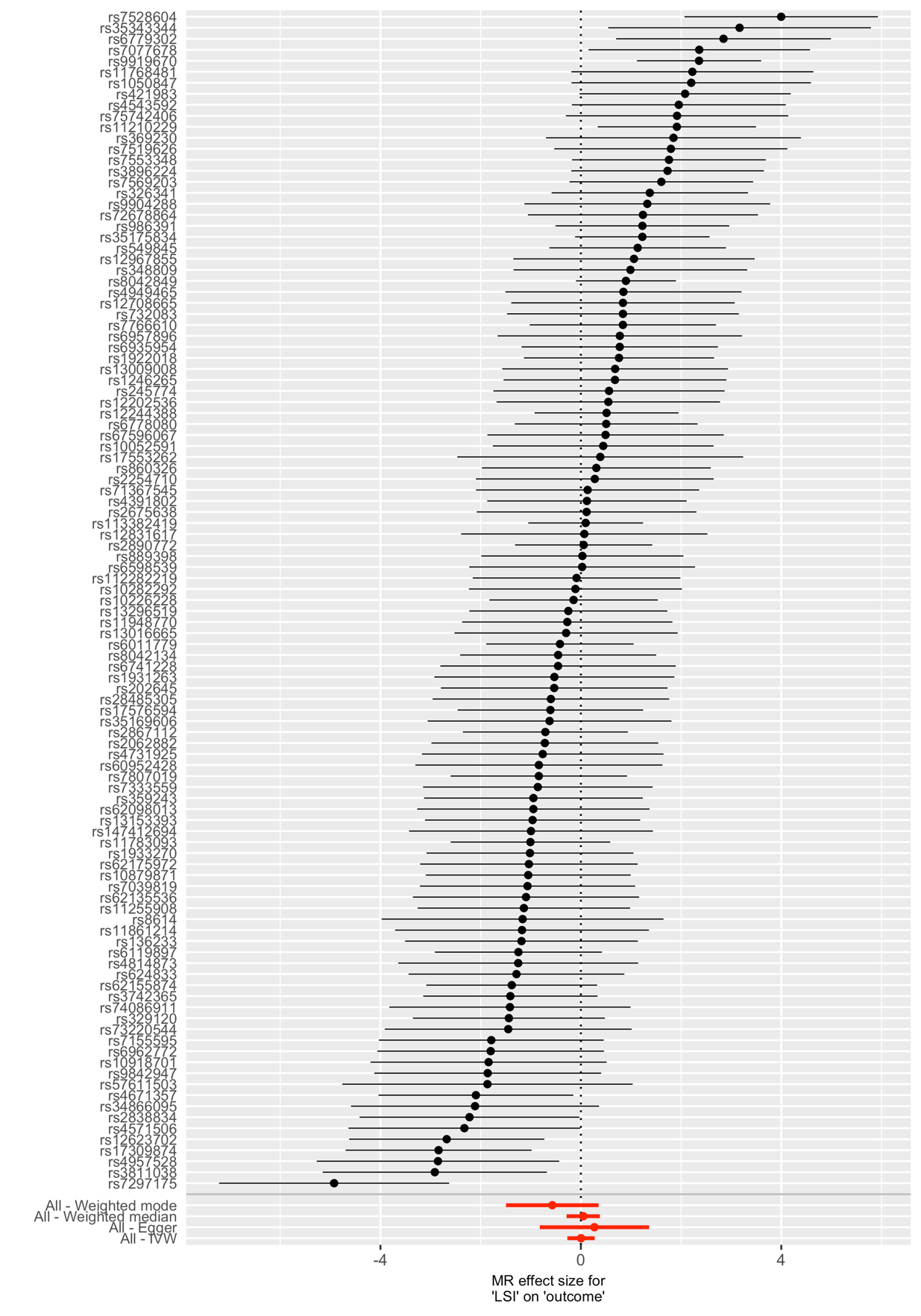


Figure S12. Leave-one-out sensitivity analysis for smoking initiation on Anorexia Nervosa

Forest plot showing inverse variance weighted (IVW) estimates after omitting each SNP one at a time. No single-SNPs were driving the effects, as all of the leave-one-out analyses were consistent with the overall estimates in red.


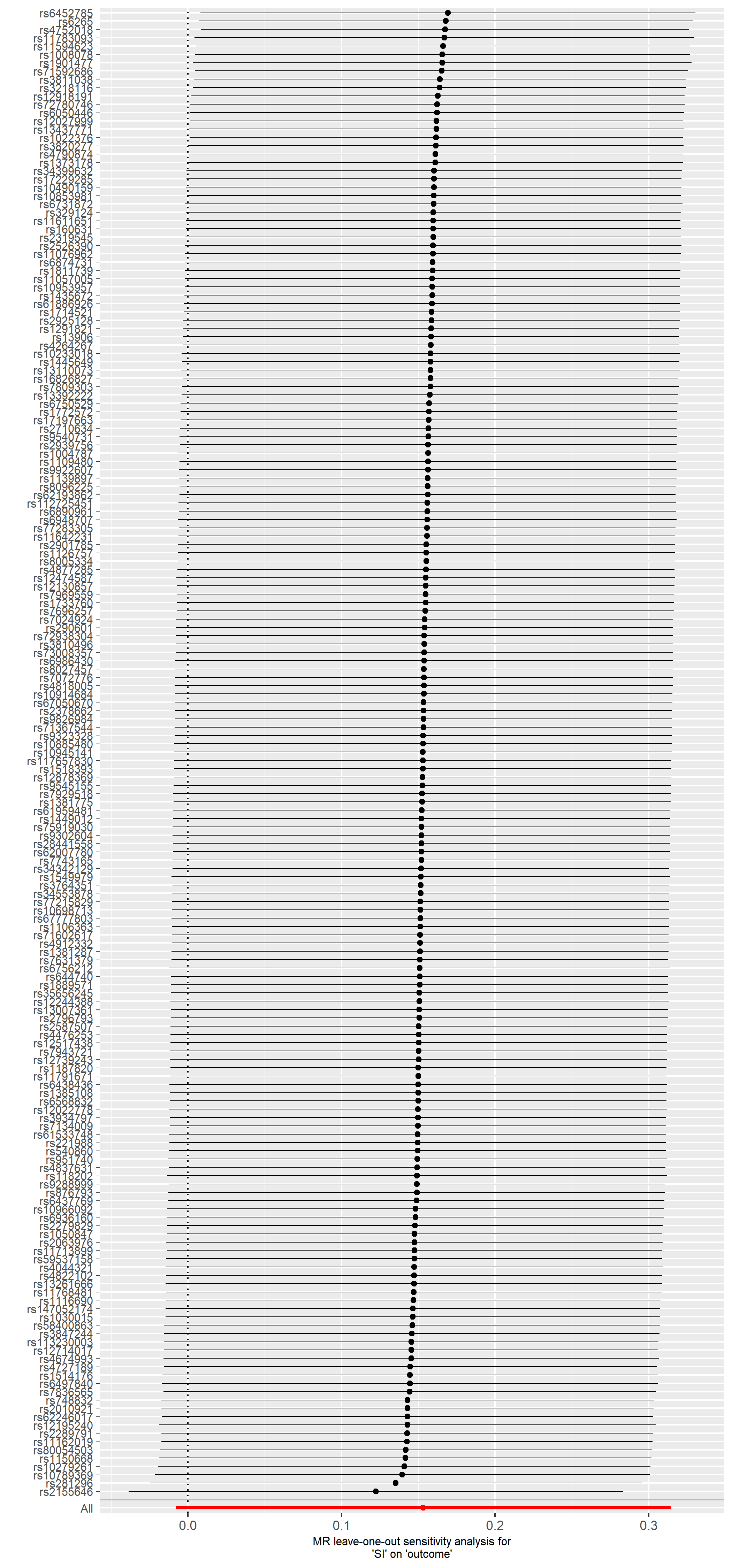


Figure S13. Leave-one-out sensitivity analysis for lifetime smoking on Anorexia Nervosa

Forest plot showing inverse variance weighted (IVW) estimates after omitting each SNP one at a time. No single-SNPs were driving the effects, as all of the leave-one-out analyses were consistent with the overall estimates in red.


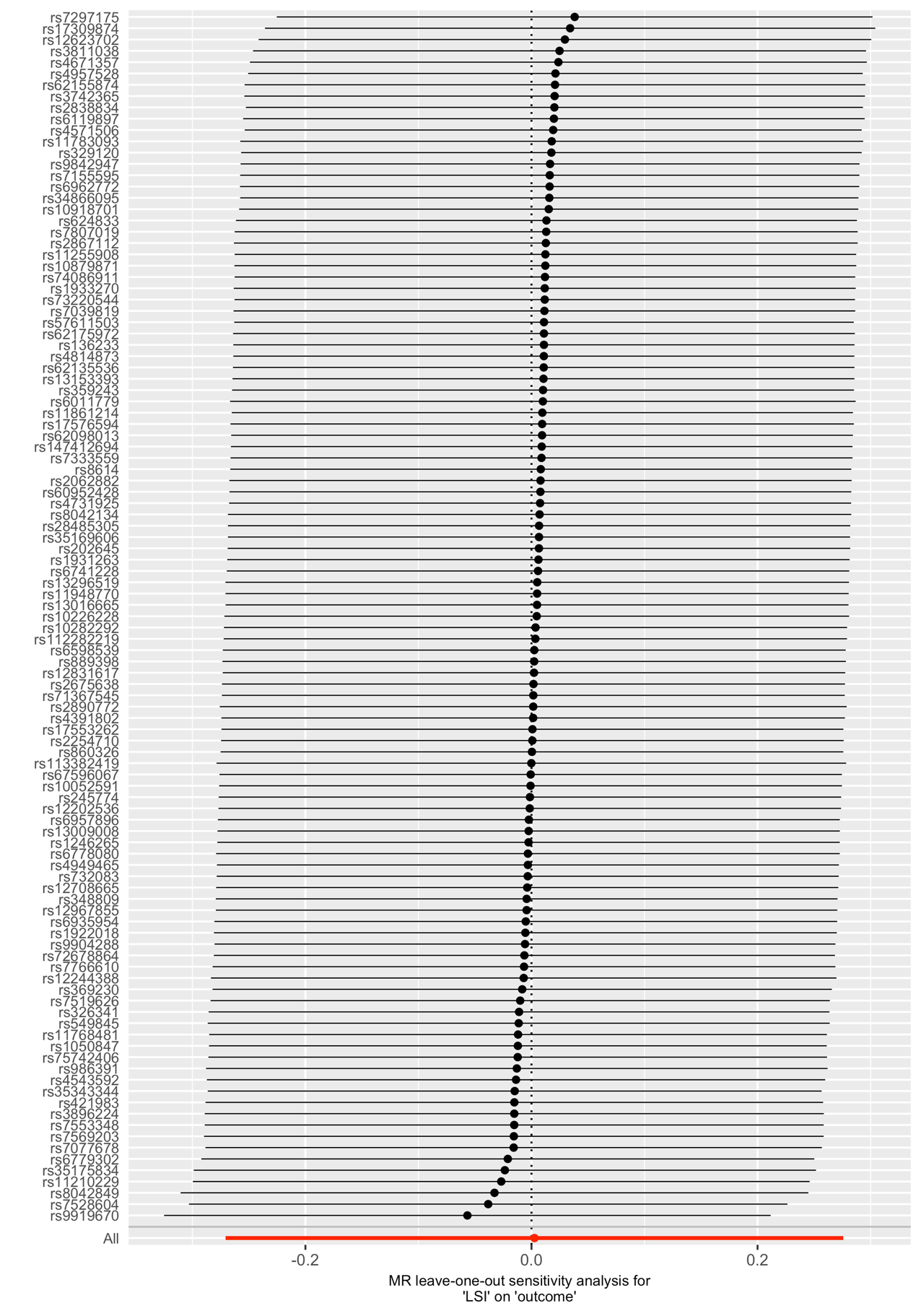


**References**

[1] L. Kann *et al.*, "Youth risk behavior surveillance--United States, 1995," (in eng), *J Sch Health,* vol. 66, no. 10, pp. 365-77, Dec 1996.

[2] C. M. Shisslak *et al.*, "Development and evaluation of the McKnight Risk Factor Survey for assessing potential risk and protective factors for disordered eating in preadolescent and adolescent girls," (in eng), *Int J Eat Disord,* vol. 25, no. 2, pp. 195-214, Mar 1999.

[3] A. E. Field, C. A. Camargo, C. B. Taylor, C. S. Berkey, S. B. Roberts, and G. A. Colditz, "Peer, parent, and media influences on the development of weight concerns and frequent dieting among preadolescent and adolescent girls and boys," (in eng), *Pediatrics,* vol. 107, no. 1, pp. 54-60, Jan 2001.

[4] R. Goodman, T. Ford, H. Richards, R. Gatward, and H. Meltzer, "The Development and Well-Being Assessment: description and initial validation of an integrated assessment of child and adolescent psychopathology," (in eng), *J Child Psychol Psychiatry,* vol. 41, no. 5, pp. 645-55, Jul 2000.

[5] "Physical status: the use and interpretation of anthropometry. Report of a WHO Expert Committee," (in eng), *World Health Organ Tech Rep Ser,* vol. 854, pp. 1-452, 1995.

[6] T. J. Cole, J. V. Freeman, and M. A. Preece, "British 1990 growth reference centiles for weight, height, body mass index and head circumference fitted by maximum penalized likelihood," (in eng), *Stat Med,* vol. 17, no. 4, pp. 407-29, Feb 1998.

[7] M. J. Brion, K. Shakhbazov, and P. M. Visscher, "Calculating statistical power in Mendelian randomization studies," (in eng), *Int J Epidemiol,* vol. 42, no. 5, pp. 1497-501, Oct 2013, doi: 10.1093/ije/dyt179.

[8] J. Bowden, F. Del Greco M, C. Minelli, G. Davey Smith, N. A. Sheehan, and J. R. Thompson, "Assessing the suitability of summary data for two-sample Mendelian randomization analyses using MR-Egger regression: the role of the I2 statistic," *International Journal of Epidemiology,* vol. 45, no. 6, pp. 1961-1974, 09/11
